# Structure, function, and applications of two novel phage recombinases from extreme environments

**DOI:** 10.1093/nar/gkag069

**Published:** 2026-02-11

**Authors:** Emma Tarrant, Isabel G Cormack, Charlotte E Hunter, Olesia Werbowy, Sebastian Dorawa, Lei Wang, Ida Helene Steen, Ruth-Anne Sandaa, Elísabet Eik Guðmundsdóttir, Bernd Ketelsen-Striberny, Anna-Karina Kaczorowska, Tadeusz Kaczorowski, Ehmke Pohl, Stefanie Freitag-Pohl

**Affiliations:** Department of Chemistry, Durham University, South Road, Durham DH1 3LE, United Kingdom; Department of Chemistry, Durham University, South Road, Durham DH1 3LE, United Kingdom; Department of Chemistry, Durham University, South Road, Durham DH1 3LE, United Kingdom; Department of Microbiology, Faculty of Biology, University of Gdansk, Wita Stwosza 59, 80-308 Gdansk, Poland; Laboratory of Extremophiles Biology, Department of Microbiology, Faculty of Biology, University of Gdansk, Wita Stwosza 59, 80-308 Gdansk, Poland; Institute of Biomedical Genetics, University of Stuttgart, Allmandring 31, 70569 Stuttgart, Germany; Department of Biological Sciences, Center for Deep Sea Research, University of Bergen, Postboks 7803, N-5020 Bergen, Norway; Department of Biological Sciences, Center for Deep Sea Research, University of Bergen, Postboks 7803, N-5020 Bergen, Norway; MATIS Ohf, Vínlandsleið 12, 113 Reykjavík, Iceland; ArcticZymes Technologies ASA, Sykehusvegen 23, 9019 Tromsø, Norway; Collection of Plasmids and Microorganisms, Department of Microbiology, Faculty of Biology, University of Gdansk, Wita Stwosza 59, 80-308 Gdansk, Poland; Laboratory of Extremophiles Biology, Department of Microbiology, Faculty of Biology, University of Gdansk, Wita Stwosza 59, 80-308 Gdansk, Poland; Department of Chemistry, Durham University, South Road, Durham DH1 3LE, United Kingdom; Biophysical Science Institute, Durham University, South Road, Durham DH1 3LE, United Kingdom; Department of Chemistry, Durham University, South Road, Durham DH1 3LE, United Kingdom

## Abstract

This study describes the identification and characterization of two new extremophilic phage recombinases, UvsX_t_ and UvsX_p_, discovered through metagenomic analysis within the Virus-X project, and explores their potential applications in biotechnology. DNA recombinases are essential for maintaining genome integrity across all kingdoms of life by facilitating homologous recombination and repairing double-stranded DNA breaks. Their capacity to bind and stabilize single-stranded DNA (ssDNA) has led to wide-ranging applications in molecular biology. UvsX_t_ and UvsX_p_ show homology with known bacterial RecA and viral UvsX recombinases, including conservation of key catalytic residues and DNA-binding motifs. Biochemical assays reveal that both enzymes exhibit superior DNA strand-exchange activity compared to *Escherichia coli* RecA. High-resolution crystal structures of UvsX_t_ (2.0 Å) and UvsX_p_ (2.6 Å) confirm a conserved RecA-like core fold, with distinct structural variation at the N-terminus responsible for oligomerization. However, in spite of their similarities, we show that neither enzyme is capable to functionally replace RecA in *E. coli*. Their remarkable thermostability and functionality across diverse chemical environments highlights their robustness for biotechnological use. Notably, UvsX_t_ enhances loop-mediated isothermal amplification of viral RNA by stabilizing ssDNA intermediates. These findings expand the repertoire of thermostable recombinases with potential utility in diagnostic applications.

## Graphical Abstract

**Graphical Abstract:**
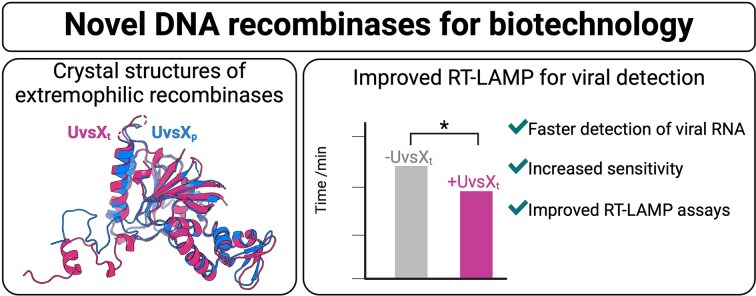

## Introduction

Recombinases are DNA-binding proteins found across all domains of life, crucial for maintenance and repair of damage to the genome. They manage this through multiple activities, mainly via homologous recombination and the formation of Holliday structures in the repair of double-strand DNA breaks [1]. Due to the essential function of these proteins, they are ubiquitous among organisms, such as UvsX in bacteriophages, RecA in bacteria, RadA in archaea, and RAD51 in eukaryotes [1, 2]. The basic architecture of these recombinases is maintained throughout evolution. They consist of a short N-terminal helical part [consisting of 34 residues in RecA from *Escherichia coli* (*Ec*RecA)] for anchoring the adjacent protomer, an ATPase core (236 residues in *Ec*RecA), and a double-stranded DNA (dsDNA)-binding C-terminal domain (CTD, 83 residues in *Ec*RecA) [3]. Although RecA is a DNA-dependent ATPase, its core functions in DNA pairing and strand exchange do not require ATP hydrolysis. However, bacterial RecAs couple several additional reactions to ATP hydrolysis that are not promoted by its archaeal and eukaryotic homologs [3]. The unique activities of RecA include: bypass of heterologous insertions in strand exchange, four-strand exchange, replication-fork regression, and an indirect helicase activity [4–7]. These reactions feature the movement of DNA branches over thousands of base pairs and/or the complete unwinding of segments of duplex DNA. RecA is therefore also a motor protein [1]. In comparison, the T4 phage recombination system is simpler, with fewer regulatory and accessory factors, making the viral orthologs ideal for recombinase studies that are likely to be conserved in higher organisms [8].

To enable recombinase activity, presynaptic protein–DNA filaments must form [3, 9, 10]. These filaments facilitate the homologous alignment of single-stranded DNA (ssDNA) and dsDNA molecules and the strand exchange that follows. Initially, recombinases non-specifically assemble around a ssDNA break, forming a filament, which consists of a right-handed helix with approximately six recombinase monomers bound per turn. Although filament without co-factors ATP and Mg^2+^ are known (inactive form), the ATP binding site is at the recombinase monomers interface, and ATP binding leads cooperatively to the active nucleoprotein filament formation [10]. The activated filament stretches the ssDNA, which is located in the axial core of the protein helix. The donor dsDNA is bound at the recombinase CTD, and the duplex is opened in this synaptic filament for dsDNA homology search and subsequent strand exchange [11, 12]. Strand repair then commences through the formation of a heteroduplex complex, which allows DNA synthesis and ligation. In addition to the ATP-binding site, there is a lytic motif [KR]X[KR] that catalyses ATP hydrolysis located near the monomer–monomer interface in this postsynaptic filament. The hydrolysis leads to the dissociation of the filament, releasing the DNA, recombinase, and ADP.

*In vivo*, many accessory proteins participate in recombination. Liu *et al.*, describe for *E. coli* T4 phage an interplay of proteins essential for recombination [13]: the recombinase UvsX, the ssDNA-binding protein Gp32 [bacterial SSB, eukaryotic recombinase polymerase amplification (RPA)], and a recombination mediator protein UvsY (bacterial RecOR, human BRCA2, eukaryotic Rad52), as well as helicases such as UvsW. These comprise a complex network of effector proteins that precisely configure recombination activities to meet cellular requirements.

Currently, *Ec*RecA is used in a range of *in vitro* techniques that involve DNA binding, DNA repair, and UV-induced mutagenesis [14–16]. Recently, the inclusion of a thermostable RecA in the polymerase chain reaction (PCR) has also been shown to minimize non-specific products [17, 18]. In assays such as RPA, recombinases aid in the stabilization of the ssDNA but also encourage the dissociation of the dsDNA, promoting the search for homology and DNA strand exchange [19, 20]. Mandated by the recent COVID-19 pandemic, work with point-of-care diagnostics has increased, with recombinases being tested to help increase the speed or specificity of potential assays, such as loop-mediated isothermal amplification (LAMP) and RPA [21, 22]. Recombinases can also be used in broader applications, such as screening libraries with DNA probes, and even for visualizing DNA nucleofilaments with electron microscopy [23, 24]. Understanding the relationship between the structure, function, and variations of recombinases could lead to optimized activities and wider applications.

The 2016–2020 Horizon 2020 Virus-X project (Viral Metagenomics for Innovation Value) involved universities, research institutes, and small and medium enterprises (SMEs) from eight countries to investigate the genomes of extremophilic viruses found in hot springs and cold deep ocean environments [25]. The ultimate goal was to discover new viral gene products for biotechnology and pharmaceutical applications. The biodiscovery pipeline included bioprospecting of water samples, followed by DNA concentration and extraction with next-generation sequencing. Newly established bioinformatic technologies resulted in an extensive database of potential proteins (>157 million open reading frames, (ORF)), where targets of potential value were selected and forwarded to protein production and subsequent functional and structural characterization [26]. Within this framework, novel phage enzymes and orthologs were identified that show improved stability due to their host environments [27, 28]. We set out to find novel recombinases within the Virus-X metagenomic database and complete their full *in vivo* functional, *in vitro* biochemical and structural characterization. In this paper, we describe the structure and function of two UvsX-type phage recombinases and assess whether these viral enzymes can replace RecA in *E. coli*. In addition, biophysical characterization of the two novel proteins was carried out to evaluate their potential as improved biotechnology enzymes.

## Materials and methods

### Cloning

Metagenomic samples collected in the Virus-X project were processed to isolate genetic material for sequencing as described previously [25]. The gene products of the UvsX proteins described here were selected from extensive genetic databases for further analysis as potentially interesting commercial targets [29]. The synthetic sequences for UvsX_t_ and UvsX_p_ were cloned in-frame into the rhamnose-inducible vector pJOE5751, resulting in the expression plasmids pLEI514 for UvsX_t_ and v_Rec_9a_5 for UvsX_p_ [30].

### Expression and purification

The plasmids were transformed into *E. coli* BL21 (DE3) competent cells, and a pre-cultures were grown overnight at 37°C in LB, supplemented with 100 µg/ml ampicillin. Each culture was used to inoculate 2 l LB-Amp, which was incubated at 37°C until an OD_600_ of 0.4–0.6 was reached. Expression was then induced by adding L-rhamnose to a final concentration of 0.2%, and the culture was incubated at 30°C overnight [30]. Cells were harvested by centrifugation at 4000 rpm for 30 min at 4°C, and the pellet resuspended in lysis buffer (50 mM Tris, pH 7.5, 0.5 M NaCl, 20 mM imidazole) with added protease inhibitor cocktail (Roche). After sonication on ice for 2–5 min (50% power on 2 × 10% cycle), the lysate was centrifuged at 20 000 RPM for 50 min at 4°C, and the resulting supernatant passed through a 0.45 µm syringe filter. The clarified supernatant was then applied to a pre-equilibrated HisTrap FF affinity column (50 mM Tris, pH 7.5, 0.5 M NaCl, 20 mM imidazole, 2 mM β-Me) and eluted over an imidazole gradient (20–500 mM) using an FPLC system (ÄKTA Pure). Proteins were then further purified by size-exclusion chromatography. The fractions were dialysed into 20 mM HEPES, pH 7.5, 300 mM NaCl, and concentrated using Amicon Ultra-15 Filter, MWCO 10 000 (Millipore USA). The purity was confirmed by sodium dodecyl sulphate–polyacrylamide gel electrophoresis. A sample of the protein was also dialysed into 10 mM ammonium bicarbonate buffer (pH 7.5) to analyse the molecular weight by ESI-MS (UvsX_t_ calculated mass = 40.803 kDa, ESI-MS base peak = 40.672 kDa [MW – Met]; UvsX_p_ calculated mass = 38.666 kDa, ESI-MS base peak = 38.660 kDa].

### Nano differential scanning fluorimetry

The thermal stability and protein aggregation of both recombinases were analysed simultaneously by nano differential scanning fluorimetry (nanoDSF) and back reflection technology [31]. Measurements were performed using a Prometheus NT.48 instrument (NanoTemper Technologies, Germany). The standard-grade glass capillaries were filled with 10 µl of UvsX_t_ or UvsX_p_ proteins (1 mg/ml in 20 mM Tris–HCl, pH 8.0, 50 mM KCl, 5% glycerol) and placed into the sample holder. The assay was run with a temperature gradient ranging from 20 to 95°C (1°C steps per minute). Protein unfolding was measured by detecting a change in the fluorescence of tryptophan and tyrosine emission wavelengths at 330 and 350 nm. Data analysis was performed using NT.Melting Control software (NanoTemper Technologies) with melting temperatures (*T*_m_) and mid-aggregation temperatures (*T*_agg_) calculated according to the manufacturer’s instructions.

### Thermal shift assays

These assays were carried out using the Durham Screens (Molecular Dimensions Inc.) [32]. Briefly, 1 ml of protein at ~1 mg/ml in 20 mM sodium phosphate, pH 7.2, 100 mM NaCl was added to 4 µl SYPRO Orange dye (5000X in dimethyl sulfoxide, (DMSA)) and 10 µl aliquoted into a 96-well PCR plate. Ten microliters of each screen solution were then added to the wells, and the plate sealed with a thermal-stable film. The plates were centrifuged at 1000 RPM briefly at 4°C before loading into a real-time PCR machine (BioRad), running the assay with a temperature gradient ranging from 25 to 95°C (1°C increase per min), and the fluorescence measured after each increment. The data were then analysed using NAMI [33].

### Microscale thermophoresis

Microscale thermophoresis was used to analyse the binding affinity between purified protein and dT_70_ oligomer with a fluorescent label [34]. Measurements were performed at 25°C in 10 mM Tris–HCl, pH 8.5, 0.5 mM MgSO_4_, 50 mM KCl, and 0.05% Tween-20, in a volume of 20 µl. Reactions were carried out with a constant amount of substrate oligomer (500 pM), titrated against decreasing concentration of proteins (50 µM–1.53 nM). After incubation for 10 min at 25°C, the reaction mixtures were transferred to standard glass capillaries. Measurements were taken using a Monolith NT.115^Pico^ instrument (NanoTemper Technologies, Germany) using 40% infrared laser power and 20% LED power. Data from a minimum of three replicate binding assays were analysed, and the equilibrium dissociation constant (*K*_d_) was determined by nonlinear fitting of the thermophoresis responses using the NT.Analysis software (NanoTemper Technologies).

### ATPase activity assay

ATPase activity of purified protein was measured according to the ATPase/GTPase Activity Assay kit (MAK113; Sigma–Aldrich) manufacturer’s instructions. First, the phosphate standards were set up as indicated in the protocol at 50, 40, 30, 20, 10, 5, and 0 µM. Second, a series of dilutions of the enzyme was set up in the Assay Buffer (40 mM Tris, 80 mM NaCl, 8 mM MgAc_2_, 1 mM ethylenediaminetetraacetic acid, pH 7.5). The sample reactions and control wells were set up according to the scheme, each test performed in at least triplicate. The reaction of protein at specified concentration with 4 mM ATP in Assay Buffer (working concentration diluted fourfold for each) was incubated for 30 min at room temperature. Reagent was added to each well to quench the reaction and incubated for a further 30 min at room temperature. Absorbance at 620 nm was read using Synergy HTX Multimode reader (BioTek). Relative OD values were normalized with background controls without ATP added. Concentrations of free phosphate [Pi] were calculated from the standard curve.

### DNA strand exchange fluorescence assays

The recombinase-assisted fluorescence assay for DNA strand exchange was conducted according to the adapted FRET method described previously [35]. The sequences of the 28-bp double-stranded probes were 5′-FAM-AAACTAATAAGATTTACAACAATTTCTC-3′ and 3′- DABCYL-TTTGATTATTCTAAATGTTGTTAAAGAG-5′ (IDT DNA). The 84-nt single-stranded target oligonucleotide sequence consisted of three replicates of the FAM probe sequence: [5′-AAACTAATAAGATTTACAACAATTTCTC-3′]_3_ (IDT DNA). Recombinases UvsX_t_ and UvsX_p_ were purified as described previously, and the RecA protein purchased from NEB. The assay was conducted in a 125 µl reaction mixture (20 mM Tris–HCl, pH 7.5, 2 mM MgCl_2_, 20 mM NaCl, 1 mM ATP), with an ATP regeneration system (3 mM phosphoenolpyruvate, 30 U/ml pyruvate kinase), 10 µM of the ssDNA target oligo, and 3 µM recombinase. To promote presynaptic filament formation, the samples were incubated at 37°C for 3 min before inducing strand exchange by addition of 125 μl mixture containing 12 mM MgCl_2_ and 3 µM dsDNA probe. The reaction was carried out in a 96-well plate reader (BioTek, Synergy H4). The fluorescence was excited at 490 nm and emission recorded at 520 nm for 30 min taking measurements every 30 s. Prior to each measurement, the plate was gently shaken at 60 rpm for 5 s to ensure evenly mixed solution. Triplicate technical repeats were taken for each recombinase with ATP at 1 mM, duplicates at 2 mM concentrations.

### *In vivo* UV sensitivity assays

The UV sensitivity was measured in BW25113 and BW25113 Δ*recA* background strains (Keio Library) containing both UvsX expression plasmids. The empty pJOE5751 plasmid was also transformed into both background strains as a control. Cultures of each strain were grown in low salt LB until reaching an A_600_ of 0.4 before chilling on ice. Serial dilutions were carried out in 10-fold steps until 10^−5^. A 10 µl spot of each dilution for each strain was spotted onto low-salt agar and allowed to fully dry before testing. The experiments were conducted in a technical duplicates with two agar plates for each UV exposure time. Irradiation was performed using a UV lamp (distance of 58 cm from a standard 15 W UV lamp with a peak output at 254 nm) for doses ranging in seconds of 0–20 J/m^2^. Plates were immediately transferred to a dark space and incubated at 30°C overnight. Assays were run in duplicate.

### RT-LAMP

The primers used were synthesized by Integrated DNA Technologies and have been previously described [36]. The 30 µl reaction volume consisted of 30 mM Tris–HCl, pH 8.8, 10 mM (NH_4_)_2_SO_4_, 50 mM KCl, 0.1% Tween-20, 8 mM MgSO_4_, 1.4 mM each of dATP, dGTP, dCTP, and dUTP, 0.8 µM of FIP and BIP primers, 0.2 µM of F3 and B3 primers, 0.4 µM of LF and LB primers, 9.6 U IsoPol® BST^+^ DNA polymerase (ArcticZymes), 8.1 U SuperScript™ IV RT (Invitrogen), 1 U Antarctic thermolabile uracil DNA glycosylase (New England Biolabs), and 5 µM Syto13 fluorescent dye (Invitrogen). MS2 RNA was added as indicated in the results; all negative template controls were prepared using nuclease-free water without the addition of RNA. Samples were incubated at 65°C for 45 min in a qPCR machine (BioRad), and the fluorescence emission was measured every 30 s. All assays were run in triplicate.

### Crystallization

Initial crystallization experiments were conducted using a range of commercially available crystallization screens set up in MRC sitting drop vapor-diffusion trays using a Mosquito crystallization robot (TTP Labtech) at room temperature. Both UvsX_t_ and UvsX_p_ crystallized under several conditions and always produced rod-shaped crystals. These conditions were further optimized, and experiments were manually set up in sitting drop trays incubated at 20°C. For UvsX_t_, the largest crystals formed with a protein solution of 6.5 mg/ml (20 mM Tris, pH 7.5, 100 mM NaCl buffer) equilibrated against 0.1 M MES, pH 6.5, 6% 1,4-dioxane, and 1.6 M ammonium sulphate. UvsX_p_ crystals formed at 2.7 mg/ml protein concentration (in 20 mM HEPES, pH 7.5, 300 mM NaCl) equilibrated against 0.12 M monosaccharides, 0.1 M Buffer System 3 (pH 8.5), and 30% v/v Precipitant Mix 2 (MORPHEUS screen, Molecular Dimensions Inc.). Crystals were harvested, cryo-protected with 25% glycerol and flash-frozen in liquid nitrogen [37].

### Data collection and structure solution

Crystallographic data were collected at Diamond Light Source (DLS, Didcot, UK) beamlines I24 (UvsX_t_) to 2.0 Å resolution and I03 (UvsX_p_) to 2.6 Å resolution using PILATUS pixel-array detectors [38, 39]. Data were reprocessed using XDS for UvsX_t_ and Xia2/Dials for UvsX_p_, followed by POINTLESS and AIMLESS as implemented in CCP4i2 [40–43]. The solution of the UvsX_t_ data was attempted using molecular replacement methods employing the crystal structure of the T4 *Enterobacteria* Phage UvsX (3IO5), despite a relatively low sequence identity of 32%. The search model was cut according to the sequence alignment with the target protein, and MR was tried with Phaser, searching for one monomer in the crystallographic asymmetric unit [44]. Phaser found a unique solution in space group P6_1_ using several different reduced search models, but always with a relatively low LLG and high *R*-values in the following REFMAC5 refinement (*R*-value 0.52/*R*_free_ 0.53) [45]. To confirm the solution, Arcimboldo-Shredder was run, yielding the same central chain and several new fragments as output [46]. After fitting and refining of the Arcimboldo output, the *R*-value/*R*_free_ dropped to 0.39/0.41. A Buccaneer run for automated model building reduced *R*-value/*R*_free_ to 0.26/0.32 (84% of the residues build) and 0.24/0.30 (96% of the residues build) [47]. Final refinement *R*-value/*R*_free_ are 0.21/0.27 with REFMAC5 for UvsX_t_ [45]. The final model contains residues HHGS from the N-His_6_ tag, as well as residues 2–160, 170–203, 221–235, 246–338, excluding residues in loop regions L1, L2, and in the dsDNA-binding regions, which were not defined in the electron density maps. One sulphate ion and water molecules are included in the model. The structure of UvsX_p_ was solved with UvsX_t_ as a search model for molecular replacement using Phaser [44]. The structure was refined with REFMAC5 to a final *R*-value/*R*_free_ 0.258/0.306, with one molecule in the asymmetric unit [45]. The final model consists of residues 6–63, 66–160, 173–202, 221–234, 245–334, and water molecules. The dynamic regions at the L1 and L2 loops (ssDNA binding) and the dsDNA-binding site were not observed in the electron density and were omitted. All model building and evaluation were performed with Coot [48]. The final models were checked using MolProbity [49]. Least-squares superpositions of Cα atoms were performed with CCP4mg [50]. Further crystallographic data are summarized in Table 1. Coordinates and structure factors have been deposited in the Protein Data Bank with accession codes 7Z3M (UvsX_t_) and 9GBG (UvsX_p_).

**Table 1.** Crystallographic data

	UvsX_t_	UvsX_p_
PDB ID	7Z3M	9GBG
Data collection		
Wavelength (Å)	0.96 857	0.970 902
Space group	P6_1_	P6_1_
Cell dimensions *a *= *b, c* (Å)	90.89, 101.44	116.75, 60.91
Resolution (Å)	41.51–2.00 (2.05–2.00)	101.09–2.60 (2.65–2.60)
No. unique reflections	32 097 (2342)	14 727 (733)
*R*_merge_	0.091 (1.823)	0.059 (1.178)
*R*_pim_	0.031 (0.626)	0.022 (0.438)
I / σ (I)	13.7 (1.3)	25.2 (1.0)
Completeness (%)	99.9 (99.2)	100.0 (100.0)
Multiplicity	9.9 (9.3)	15.6 (16.1)
CC½ (%)	0.999 (0.573)	1.000 (0.871)
**Refinement**		
*R*_work_	0.204	0.258
*R*_free_	0.251	0.306
No. protein atoms	4384	3269
No. water molecules	35	11
No. sulfate molecules	1	–
Average *B*-factors (Å^2^)		
Protein	61.1	102.7
R.m.s. deviations		
Bond lengths (Å)	0.0094	0.0061
Bond angles (°)	1.605	1.766
Ramachandran plot (%)	96.3/3.1/0.7	91.0/8.3/0.7

Data collection and refinement statistics of UvsX_t_ and UvsX_p_. Values in parentheses are for the highest resolution shell.

## Results

### Bioinformatic analysis

The proprietary Virus-X database was used to identify novel recombinase homologs through a BLAST search [25, 51]. Metagenomic samples collected across a wide range of environmental conditions were analysed to identify recombinases with potential use in biotechnology. Genetic material of two potential recombinases was discovered that showed similarity to viral UvsX, as indicated by sequence alignments. The genetic information of the first target (UvsX_t_) was collected from phages colonizing Icelandic hot springs and geysers with a temperature range of 62–88°C and a pH range from 5 to 9. In contrast, the second target (UvsX_p_) was harvested in Norwegian Arctic seawater at a depth of 1000 m and a temperature of 0.4°C. From this, we termed the first, thermophilic protein UvsX_t_, and the second, psychrophilic protein UvsX_p_.

**Figure 1.**
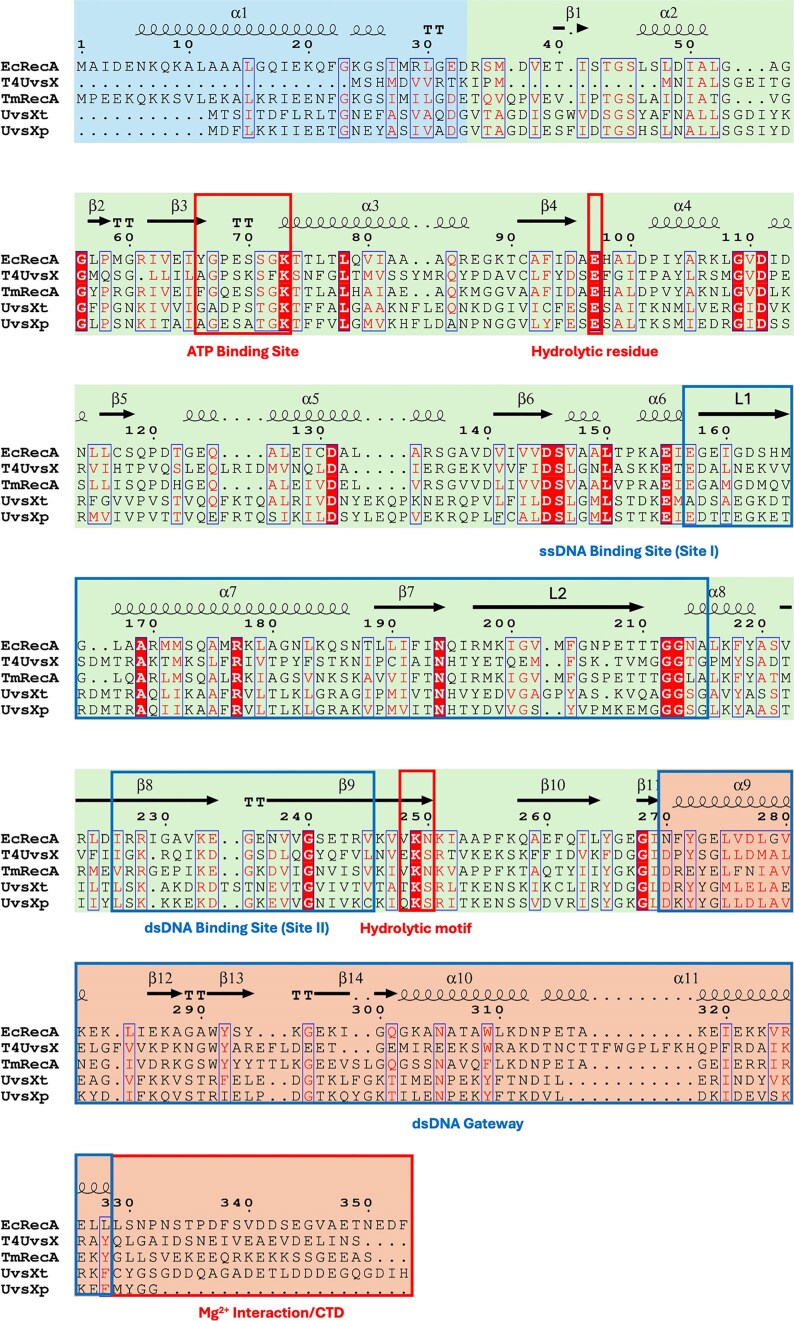
Sequence alignment of UvsX_t_ and UvsX_p_ with *E. coli* RecA, T4 UvsX, and thermostable *Thermotoga maritima* RecA. The secondary structural elements and amino acid numbers of *Ec*RecA (PDB 2reb [56]) are reported above the alignment. Domain information is provided by coloured blocks; the N-terminal domain is shown as blue, the central core ATPase domain is shown as green, and the C-terminal domain as orange. Other important regions and residues are indicated in boxes; red boxes indicate enzymatically important regions, and blue boxes are structurally important regions [3].

Initially, the protein sequences of interest were subjected to extensive databank searches [29]. These results show that UvsX_t_ has a sequence identity of 56% to uncultured Mediterranean phage uvMED UvsX (GenBank BAR35141.1). Whereas UvsX_p_ had a higher sequence identity of 88% to *Candidatus marinimicrobia* bacterium (GenBank PCH60146.1) and 76% to *Pelagibacter* phage HTVC008M (GenBank YP_007517990.1). Sequence alignment employing BlastP finds a sequence identity between our UvsX proteins and bacterial RecA in the range from 21% to 27% [51]. The *E. coli* T4 phage UvsX, which represents the only UvsX 3D structure in the Protein Data Bank, shares a sequence identity of only 32% with both of our UvsX proteins [52]. Plenty of work has been conducted on *Ec*RecA to identify functional residues and regions. Therefore, *Ec*RecA was aligned with T4 UvsX and our UvsX proteins using ESPript3 (Fig. 1) [3, 11, 53–55]. Sequence alignment of UvsX_t_ and UvsX_p_ reveals a sequence identity of 58% and sequence similarity of 78% between the two proteins.

### Stability assays

**Figure 2.**
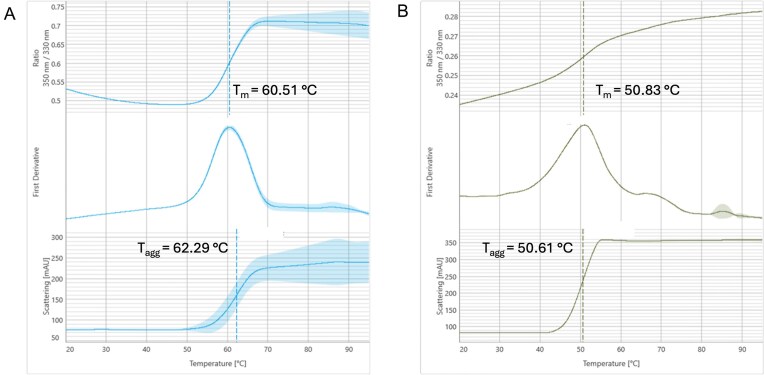
Analysis of (**A**) UvsX_t_ and (**B**) UvsX_p_ thermal stability by nanoDSF. Changes in the fluorescence ratio (350/330 nm) and the corresponding first derivative are shown in the top and middle panels, respectively. Thermal aggregation analysis of the protein is shown in the bottom panel. Vertical dashed lines in the figure indicate thermal unfolding *T*_m_ and aggregation transition *T*_agg_.

The stability of our UvsX proteins was analysed to assess temperature and salt robustness over a wide range of conditions. Thermal shift assays (TSA) were conducted with the Molecular Dimensions Durham Screens® to identify optimal buffer conditions and stabilizing or potentially incompatible components for future use as a biotechnological reagent [32]. In parallel, the thermal stability of the proteins was determined using the nanoDSF method. Analysis of the changes in the fluorescence ratio (350/330nm) and the corresponding curve indicated that *T*_m_ of UvsX_t_ is 60.5°C (±0.0°C), with *T*_agg_ at 62.3°C ± 0.3°C (Fig. 2A). The TSAs aligned well with this, as UvsX_t_ was stable until 61.1°C in phosphate buffer. The melting temperature could be increased up to 71.4°C with the addition of several salts and osmolytes (Supplementary Fig. S1). The nanoDSF experiments for UvsX_p_ showed slightly lower stability with *T*_m_ at 50.8°C (±0.1°C), and *T*_agg_ values at 50.6°C ± 0.0°C (Fig. 2B). TSAs utilizing the Durham screens for buffer optimization resulted in a *T*_m_ of 40.2°C (Supplementary Fig. S2). A wide range of conditions stabilize UvsX_p,_ as can be seen in the mostly blue (indicating higher than initial *T*_m_) tables in Supplementary Fig. S2. Especially in the Durham Salt Screen, under conditions containing malonate, a jump in *T*_m_ of up to 64.3°C could be measured. This protein shows a high stability across a very broad chemical range. Summarizing, the stability assays, the *T*_m_ of UvsX_t_ is higher (nanoDSF 60.5°C, TSAs 61.1°C) compared to UvsX_p_*T*_m_s (nanoDSF 50.8°C, TSAs 40.2°C), which agrees with UvsX_t_ coming from a thermophilic organism and UvsX_p_ DNA being harvested in a cold environment. Each protein has individual advantages for potential biotech uses. UvsX_t_ can be used at overall higher temperatures and is stable across various conditions; there are few conditions that had a severe negative effect on protein stability. In comparison, although UvsX_p_ has a lower *T*_m_, the protein can remain stable over a wider range of compounds that could be present in assay solutions.

### UvsX_t_ and UvsX_p_ functional characterization

**Figure 3.**
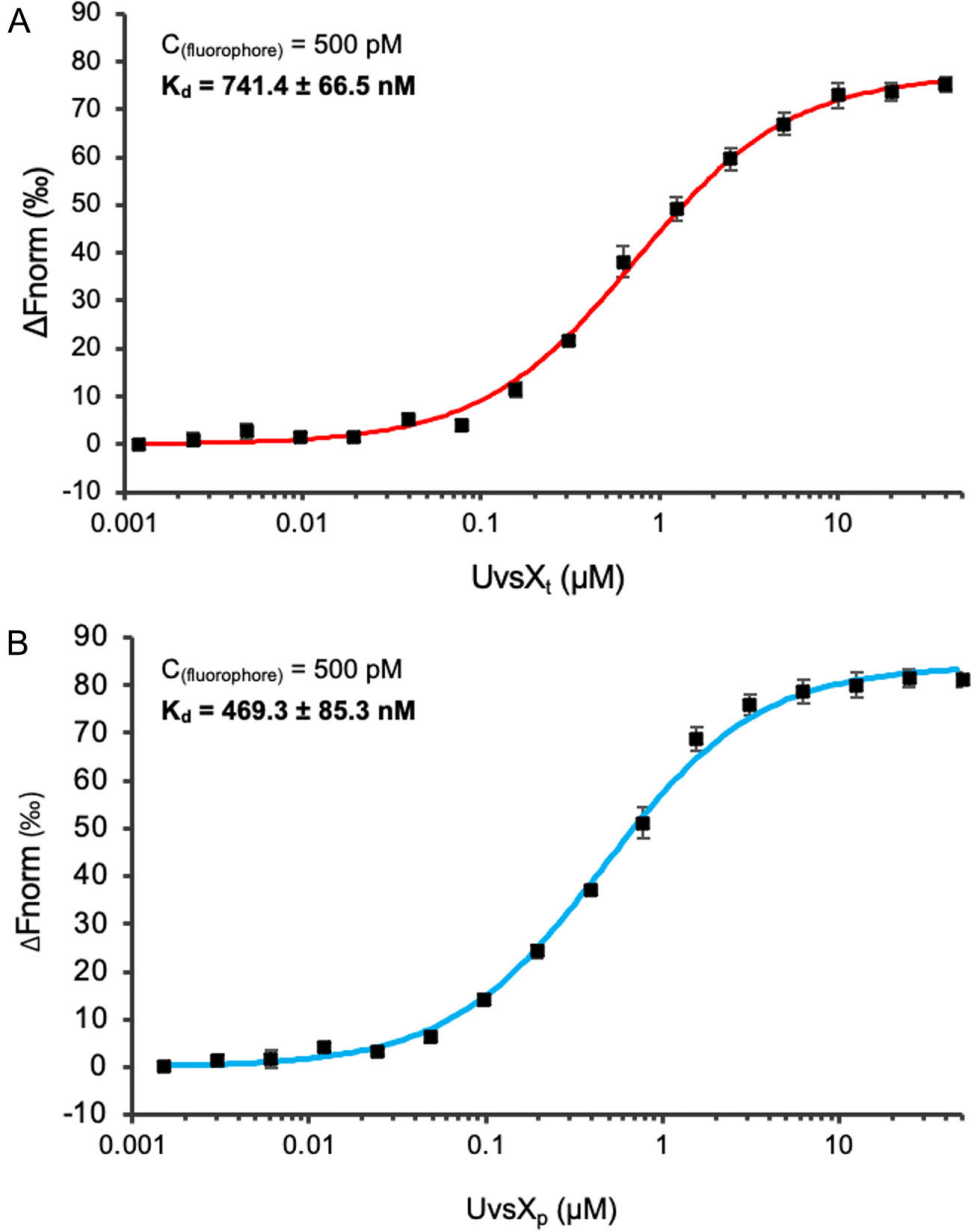
Kinetics of (**A**) UvsX_t_ and (**B**) UvsX_p_ binding to Cy5-labeled oligonucleotide (dT)_70_ as determined by microscale thermophoresis. Protein–DNA interaction was monitored by titrating proteins from 50 µM to 1.53 nM against 0.5 nM Cy5-labeled DNA. Measurements were performed at 25°C in 10 mM Tris–HCl, pH 8.5, 5 mM MgSO_4_, 50 mM KCl, and 0.05% Tween-20.

To evaluate the usability of the novel proteins, the function needed to be assessed. The DNA-binding ability of the proteins was first studied, followed by experiments to evaluate the strand-displacement activities. Using a fluorescently tagged oligonucleotide dT_70_ in microscale thermophoresis experiments, the binding affinities (*K*_d_) of UvsX_t_ and UvsX_p_ to unspecific ssDNA were determined to be 741 ± 66 nM and 469 ± 85 nM, respectively (Fig. 3).

**Figure 4.**
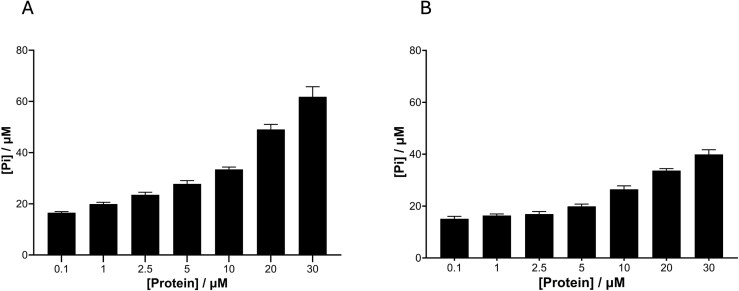
ATPase activity of (**A**) UvsX_t_ and (**B**) UvsX_p_. ATPase activity is displayed as production of phosphate detected using ATPase/GTPase Activity Assay kit (MAK113; Sigma–Aldrich), against a standard curve.

In order to show the ATPase activity, a standard assay was used. Both proteins display the expected concentration-dependent ATPase activity (Fig. 4), comparable to *Ec*RecA. UvsX_t_ showed a slightly, but statistically significant, higher activity compared to UvsX_p_, in particular at higher protein concentrations. These results establish both proteins as ATPases.

**Figure 5.**
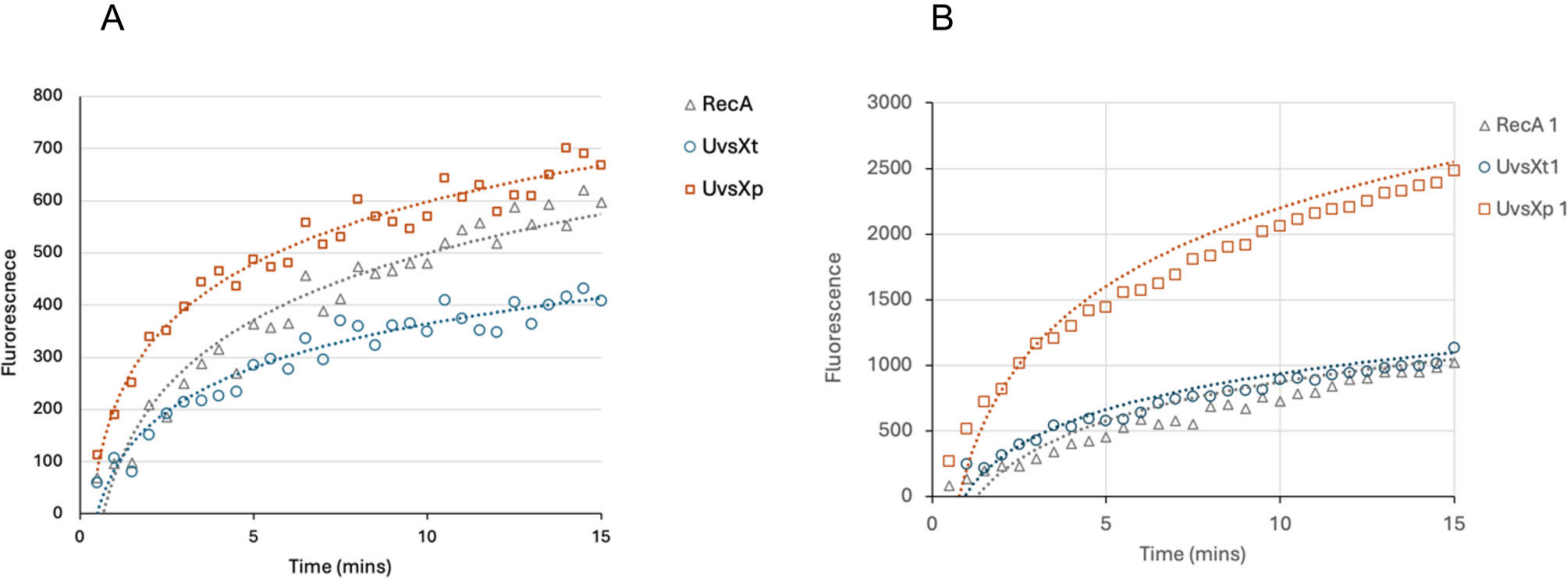
The real-time monitoring of DNA strand displacement reaction promoted by UvsX_t_ and UvsX_p_. *Escherichia coli* RecA protein (New England Biolabs) was used as a control. Two different ATP concentrations (A – 1 mM, B – 2 mM) were used. Data show absolute fluorescence values.

DNA-strand exchange fluorescence assays were performed using a dark-quenching method [35]. The 28-mer double-stranded oligo in this experiment carries two labels: 5′FAM on the first strand and 3′DABCYL on the complementary strand. In the absence of any recombination reaction, DABCYL quenches the FAM fluorescence. A single-stranded 84-mer oligo with a triple-repeat sequence was incubated with the recombinase to form presynaptic filaments in the presence of ATP. The presence of multiple contact points promotes the incorporation of presynaptic filaments into the incoming dsDNA oligo by homologous recombination, leading to the switching of pairing and strand displacement. The release of the fluorescently labelled oligo depends on the rate of the strand exchange reaction. The kinetics of strand displacement are shown in Fig. 5 for UvsX_t_ and UvsX_p_, using *Ec*RecA as a control. Initially, assays were conducted at low physiological concentration of 1 mM ATP (Fig. 5A). All proteins display the expected exponential rise in fluorescence, plateauing over the time course of the experiment. While UvsX_t_ appears to have slightly lower activity compared to *Ec*RecA, UvsX_p_ shows significantly higher activity. This effect is even more pronounced at 2 mM ATP, where UvsX_p_ shows far larger activity compared to its orthologs (Fig. 5B). Higher ATP concentrations are known to stabilize presynaptic filaments, promoting more efficient homology search and strand invasion [57–61]. Single-molecule experiments have shown that ATP-bound RecA filaments are longer and more stable, whereas ATP hydrolysis triggers filament compression and destabilization, leading to disassembly and strand reannealing. We suggest that the increased ATP concentration in our assay may enhance filament stability and nucleation rates, and prevents premature disassembly, thus accelerating strand exchange.

### UV sensitivity assay in *E. coli*

**Figure 6.**
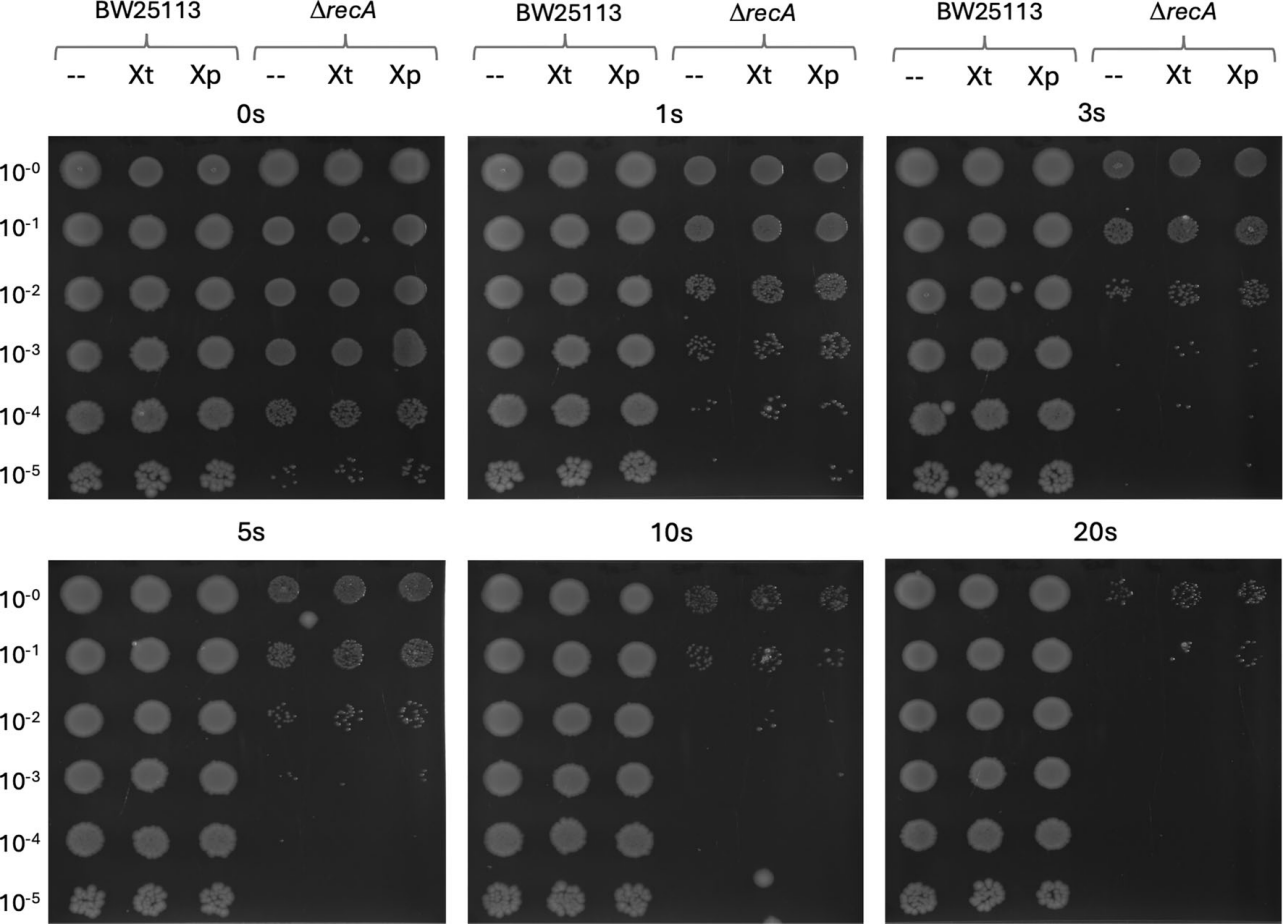
UV sensitivity assays comparing wild-type *E. coli* (BW25113) with the Δ*recA* knock-out strain. Column 1 and 4 labelled ‘–’ in each plate refer to a control containing an empty plasmid, ‘Xt’, and ‘Xp’, respectively, refer to the same plasmid with the gene. Exposure time is given from 0 to 3 s in the first, and 5 to 20 s in the second row of plates. Plates were imaged after approximately 18 h.

To determine whether our viral recombinases can functionally replace the bacterial RecA, we compared the UV sensitivity of wild-type *E. coli recA^+^* with a Δ*recA* knock-out strain complemented with *uvsX_t_* and *uvsX_p_* containing plasmids (Fig. 6). Plates were exposed to increasing rates of UV-radiation from 0 to 20 s in a serial 10-fold dilution in each row of each plate. The three columns on each plate with the wild-type (BW25113 *recA^+^*) demonstrate that compared to the Δ*recA* knock-out in the fourth column, RecA is required to restore growth to similar levels. The addition of *uvsX_t_* or *uvsX_p_* containing plasmids shows no effect on the wildtype *E. coli* strain. However, adding these plasmids to the Δ*recA* knock-out shows a small but significant improvement in growth recovery (see columns 5 and 6 in each plate). Clearly, these viral enzymes are not capable of fully rescuing bacteria with the Δ*recA* knock-out from UV-damage.

### Recombinase function in RT-LAMP

Due to the recent SARS-CoV-2 pandemic, substantial research has been conducted on designing simple but accurate *point-of-care* tests [62]. One such assay is reverse transcription loop-mediated isothermal amplification (RT-LAMP), which amplifies DNA at a constant temperature following initial reverse transcription of the starting RNA material, offering an advantage over conventional PCR-based diagnostics that require thermocycling [63, 64]. However, RT-LAMP assays can have reduced sensitivity and specificity. As recombinases bind ssDNA to induce binding and strand exchange, the addition of these enzymes could confer higher sensitivity and specificity. We tested the effect of UvsX_t_ rather than UvsX_p_, as nanoDSF and TSA results indicate it would better withstand the assay temperature of 65°C.

**Figure 7.**
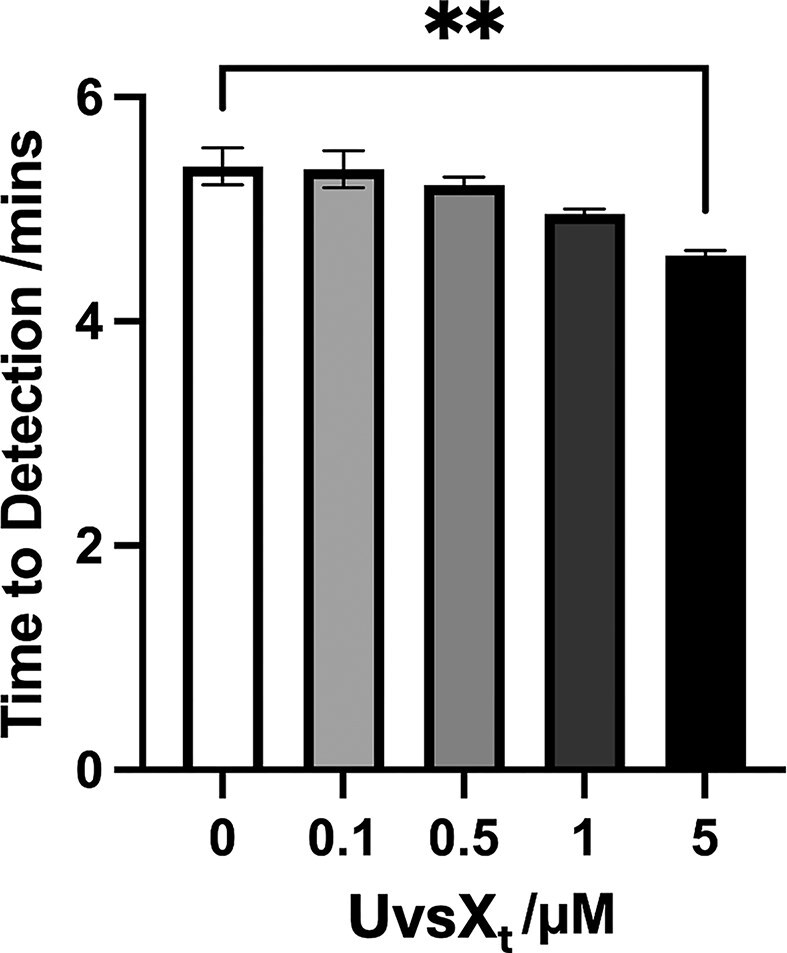
Addition of UvsX_t_ to RT-LAMP reactions results in faster detection of target RNA. When 5 µM of UvsX_t_ was used, time to results was significantly reduced compared to no addition of UvsX_t_.

**Figure 8.**
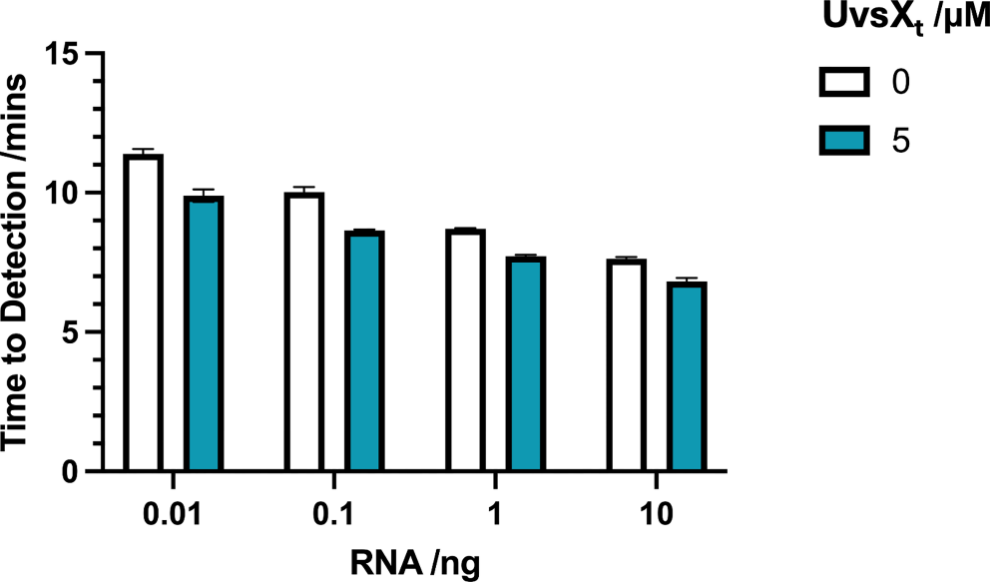
Detection time as a function of RNA amount. Addition of 5 µM of UvsX_t_ to RT-LAMP enhances the sensitivity, allowing detection of lower quantities of starting RNA material.

The addition of UvsX_t_ was initially tested across a concentration range of 0–5 µM (Fig. 7). The UvsX_t_ concentration in the RT-LAMP reaction mixture exhibited a positive correlation with amplification efficiency. Addition of 5 µM of UvsX_t_ results in a reduction in the detection time for 10 ng of RNA (*M *=4.6 mins, SD = 0.04) compared to no addition (*M* = 5.4 mins, SD = 0.17), *t*(4) = 8.02, *P* = .0013. For this reason, the effect of 5 µM of UvsX_t_ on the sensitivity of RT-LAMP was tested using decreasing quantities of starting RNA material (Fig. 8). 0.01–10 ng of RNA were added to RT-LAMP reactions, and the time taken to detect each was analysed. Overall, the addition of 5 µM of UvsX_t_ results in faster detection times at each mass of RNA tested, indicating an increase in assay sensitivity. A two-way analysis of variance was performed to analyse the effect of UvsX_t_ addition on the time to detection of different starting masses of RNA. Simple main effects analysis shows that 5 µM UvsX_t_ has a statistically significant effect on time to results at each mass of RNA, proving that UvsX_t_ increases RT-LAMP sensitivity.

The effects of UvsX_t_ were also tested with the addition of ATP (4 and 40 mM), but no fluorescence and therefore no amplification were observed. Since ATP binding induces the high-affinity conformation of RecA-family recombinases, we suggest that the subsequent slow dissociation of UvsX_t_ from ssDNA during the reaction hinders RT-LAMP activities by blocking the actions of the strand-displacing DNA polymerase.

### Structure determination of UvsX_t_ and UvsX_p_

**Figure 9.**
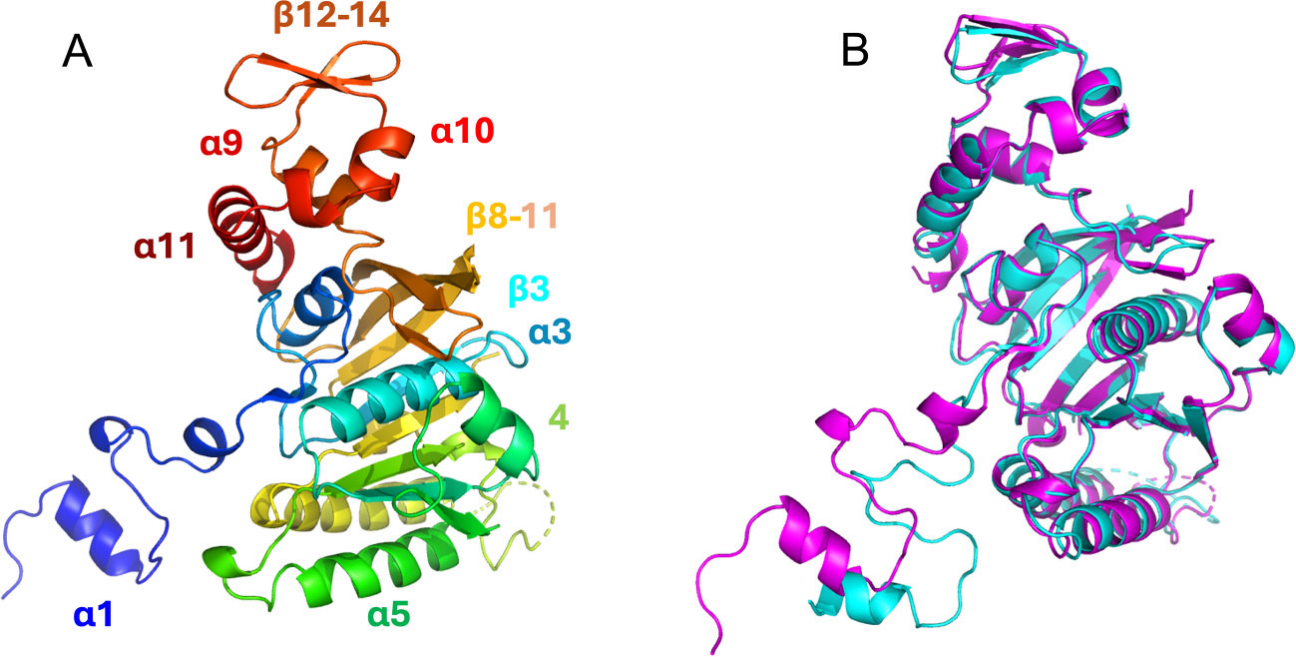
(**A**) Ribbon diagram of the UvsX_t_ structure (PDB 7Z3M) in rainbow colours from N-terminus (blue) to C-terminus (red). (**B**) Superposition of UvsX_p_ (PDB 9GBG) on UvsX_t_ using PyMOL. UvsX_t_ in magenta, UvsX_p_ in cyan.

The crystal structures of UvsX_t_ and UvsX_p_ were determined using X-ray crystallography to a resolution of 2.0 Å and 2.6 Å, respectively. Both adopt a RecA-like fold (Fig. 9A). The highly flexible N-terminal domain of the protein is formed by the α1-helix and a short β-α-β motif (Supplementary Fig. S3). The core domain consists of a five-stranded parallel β-sheet with four α-helices on one side (α5, α6, α7, α8) and α3, α4 on the other side. Joined through α-helix 8 is an adjacent four-stranded antiparallel β-sheet and the C-terminal domain. The C-terminus comprises another, less extended three-stranded antiparallel β-sheet and three neighbouring α-helices (α9–α11). A least-squares superposition of the two crystal structures (Fig. 9B) shows that the main difference is the different orientation in the flexible N-terminal regions. These parts of the structures are also involved in crystal-packing interactions; therefore, this effect might be due to crystallization conditions. The core of the UvsX structures are highly conserved (RMSD 0.90 Å for 268 residues) [50].

**Figure 10.**
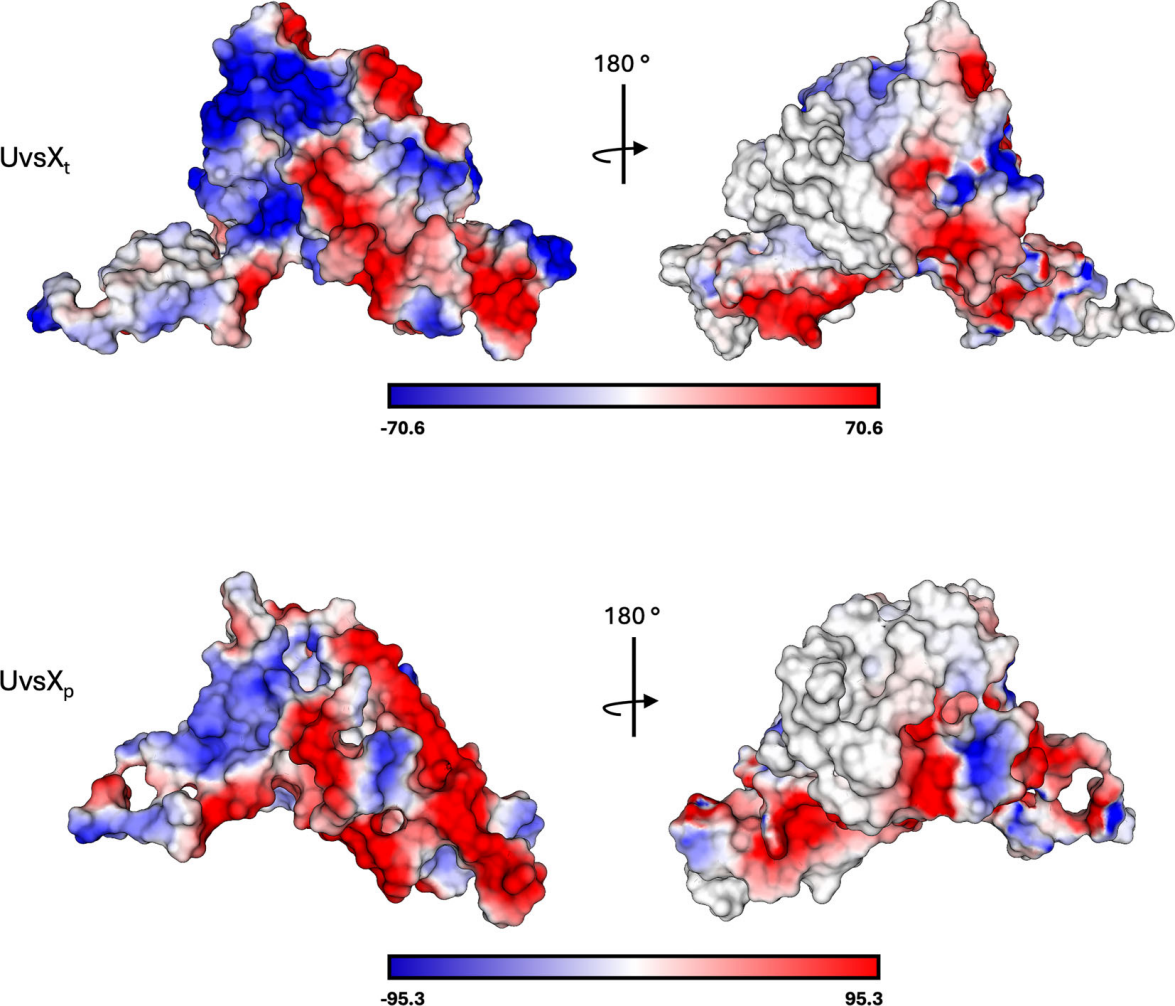
Electrostatic surface potentials of UvsX_t_ and UvsX_p_, where colour intensity indicates the strength of potential.

Further structural analysis dissected the basis for the opposite characteristics of UvsX_t_ and UvsX_p_. The ProteinTools webserver was used to analyse hydrophobic clusters within each protein, which revealed denser hydrophobic clustering within the core of the protein [65]. This promotes tighter packing of the UvsX_t_ hydrophobic core and increases the rigidity of its monomers compared to UvsX_p_, enabling them to withstand increased temperatures. Additional analysis revealed further differences between the two proteins in their electrostatic surface potential (Fig. 10). Both recombinases have charged regions on their surface, but UvsX_p_ has larger and more positively charged regions. This is proposed as a strategy for cold adaptation to increase the affinity of UvsX_p_ for negatively charged ssDNA at low temperatures, as has been observed in other psychrophilic proteins [66–68].

The salt bridge networks within each crystal structure were also revealing a 100% increase in salt bridge networks for UvsX_t_ in comparison to UvsX_p_ (4 versus 2). This was also observed when compared to the mesophilic T4 UvsX [69]. More hydrogen bond networks are also found in UvsX_t_, frequently in loop regions, which significantly reduces their flexibility. This rigidity is also reinforced by salt bridges present in loop regions, contributing to the increased thermostability of UvsX_t_ over UvsX_p_.

### Recombinase–ssDNA modelling

Initially, AlphaFold3 was employed to model the interactions between UvsX_t_ , UvsX_p_, and ssDNA [70]. The predicted protein structures exhibited a high degree of accuracy with our solved crystal structures. This was expected based on their strong sequence and structural homology to previously characterized recombinases available in the PDB [52]. However, the modelled protein–ssDNA complexes revealed a plethora of interactions that are not feasible, with ssDNA positioned too close to the protein surface (data not shown).

**Figure 11.**
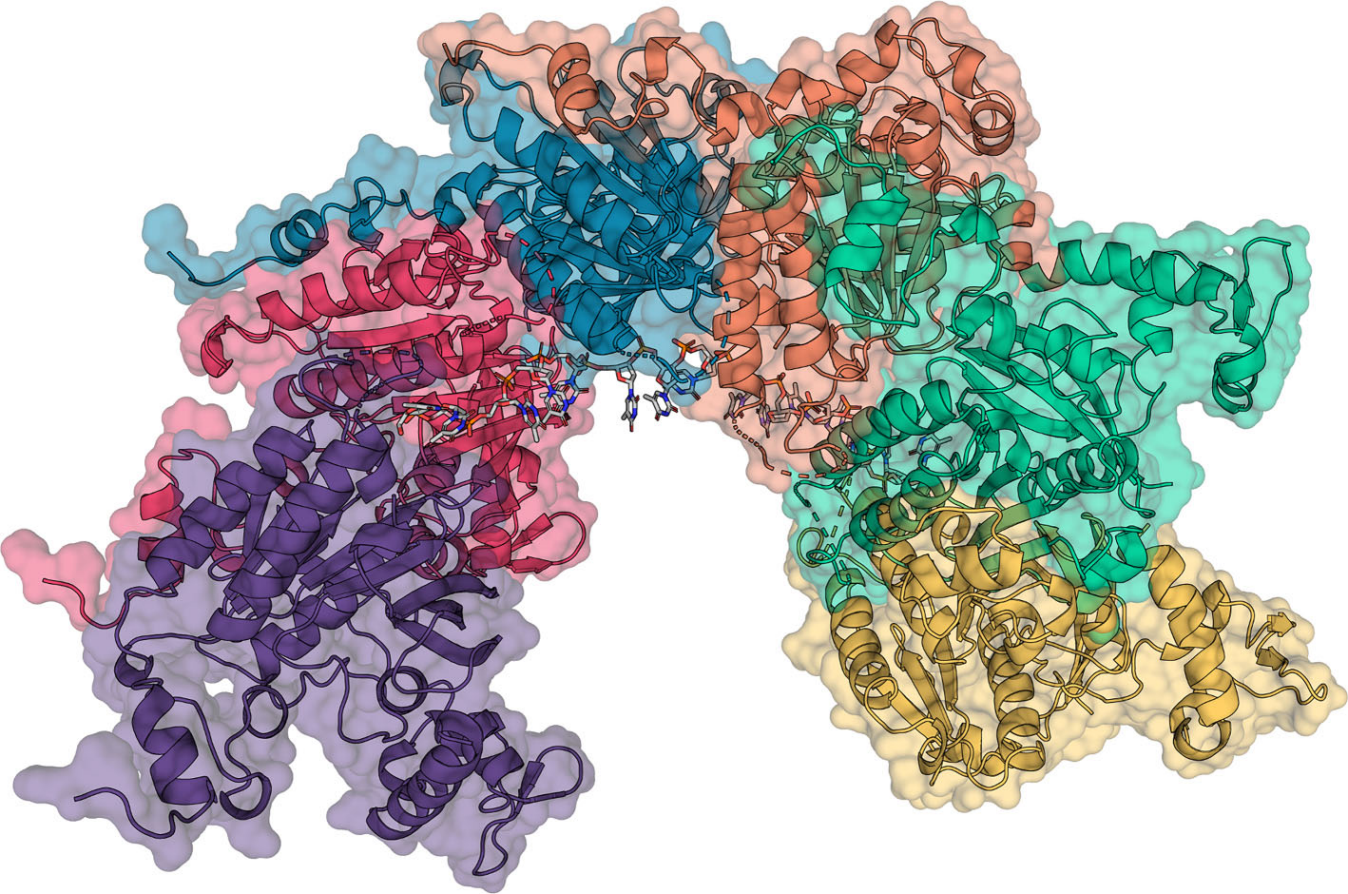
Model of UvsX_t_ helical filament along dT_18._ The six UvsX_t_ protomers are coloured purple, red, blue, orange, green, and yellow. DNA is represented by a stick model with carbon atoms in grey, nitrogen in blue, oxygen in red, and phosphorous in magenta.

Therefore, a model of a UvsX_t_ and ssDNA filament was created by aligning UvsX_t_ monomers with a RecA–ssDNA structure (PDB: 3CMU) (Fig. 11) [10]. Alignments were performed using the conserved core domains of the monomers only, as the original RecA–ssDNA structure comprised a non-native protein construct joining monomers in series. This model shows how UvsX_t_ likely binds ssDNA with its central core ATPase domain, likely by L1 and L2 if these are present in the structure.

**Figure 12.**
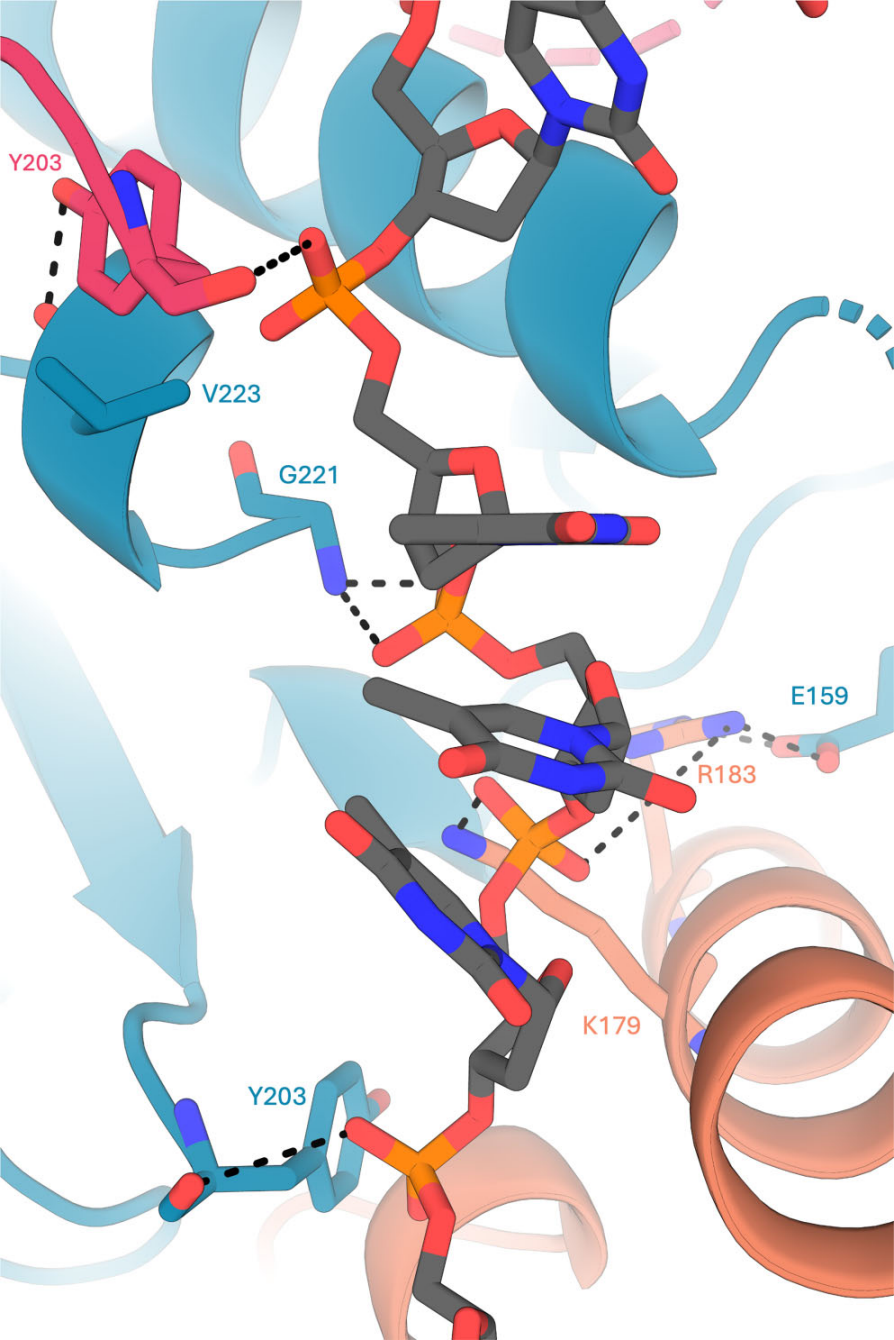
Close-up of specific protein–DNA interaction in the model. UvsX_t_ protomers are suggested to bind nucleotide triplets using three protomers. The central nucleotide is bound by UvsX in blue, with the UvsX_t_ molecule binding the 5′ nucleotide in pink, and that binding the 3′ nucleotide in orange. Hydrogen bonds are indicated with black dotted lines.

Three UvsX_t_ molecules bind each nucleotide triplet. By comparing the model to the original RecA–ssDNA filament, putative residues mediating ssDNA binding by UvsX_t_ were identified (Fig. 12). There are fewer hydrogen bonds present in the model in comparison to the RecA–ssDNA structure, likely due to key residues absent from the structure in the unresolved L1 and L2 regions. Residues in these regions likely play a role in DNA binding, as seen for other RecA-family proteins.

## Discussion

In this study, we describe UvsX_t_ and UvsX_p_ as novel phage recombinases from extreme environments [25]. *In vivo*, recombinases are crucial in the process of repairing DNA damage through homologous recombination. They are also employed in various *in vitro* assays, such as PCR and RPA, where they can stabilize transient DNA species and promote DNA strand exchange by promoting dissociation of dsDNA. Therefore, novel recombinases contribute to the optimization and development of biotechnological applications. Particularly, enzymes with thermal stability and activity over a wide range of chemical conditions are in demand.

Extensive sequence analysis identified several orthologues with putative functions, and thus potential applications in biotechnology. Once identified as potential recombinases, we conducted further sequence analysis on UvsX_t_ and UvsX_p_ to confirm their similarity to known bacterial RecAs and viral UvsXs with respect to enzymatic key functional residues and ligand binding motifs. A comparison of our protein sequences against those of *E. coli* RecA and T4 phage UvsX was conducted, as RecA properties are well-understood, while UvsX is a closer homolog of our proteins. Between all sequences, there was identity, especially at essential residues such as the hydrolytic residue (E96) and for Mg^2+^ binding (D145). Larger regions of functional importance, such as DNA-binding sites and the dsDNA gateway, show strong similarity in producing the same charges and polar effects. From these alignments, the structure and function of our novel UvsX-like proteins are significantly alike.

**Figure 13.**
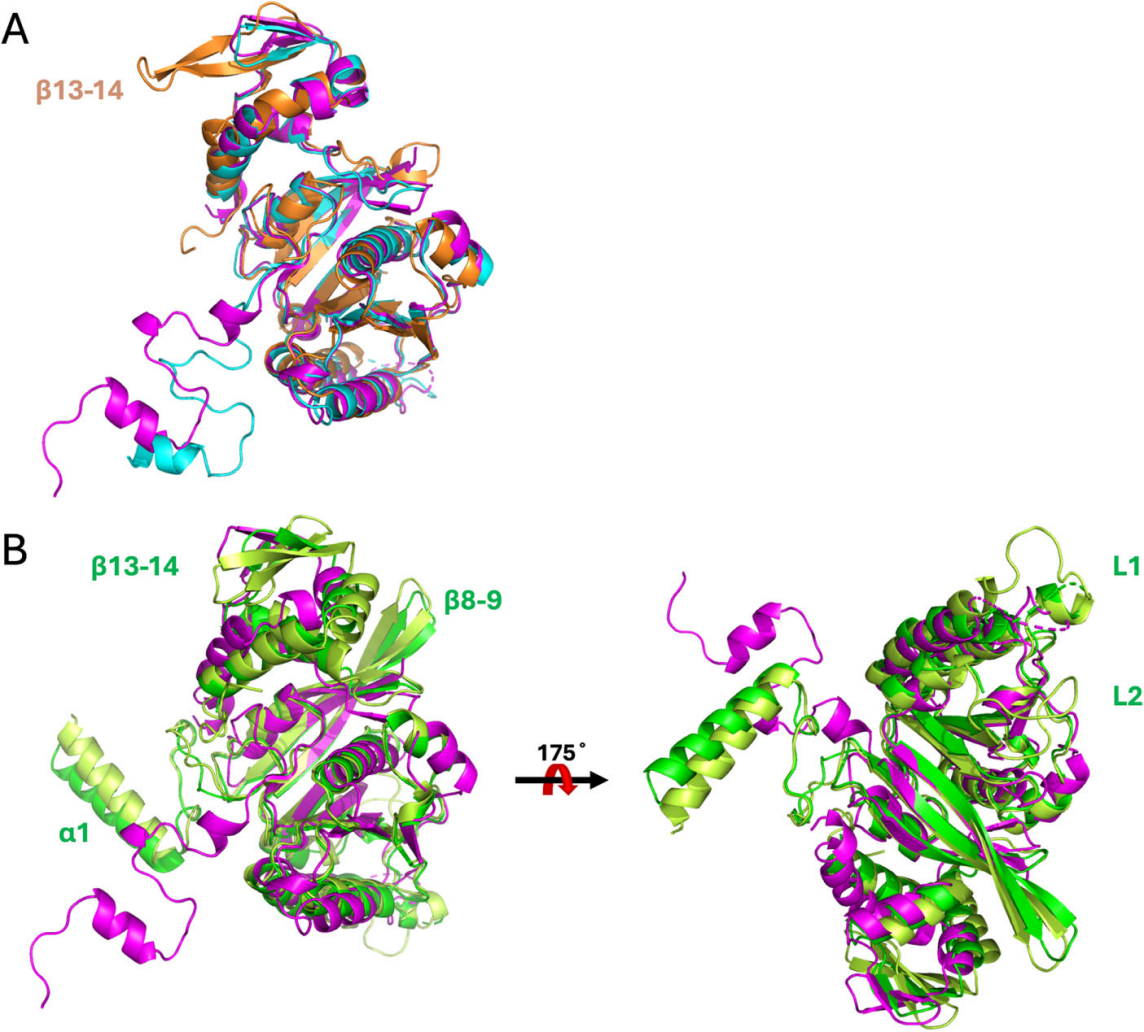
(**A**) Superposition of T4 UvsX (PDB 3IO5) on UvsX_t_ (magenta) and UvsX_p_ (cyan). T4 UvsX in orange. (**B**) Superposition of *E. coli* RecA (PDB 2REB, green) and *T. maritima* RecA (PDB 3HR8, lime) on UvsX_t_ (magenta).

To confirm the recombinase classification, crystallographic studies were carried out on the novel UvsX proteins, resulting in two crystal structures at 2.0 Å (UvsX_t_) and 2.6 Å (UvsX_p_) resolution, which show both a RecA-like fold. The Vector Alignment Search Tool VAST+ identifies similar 3D structures to the new UvsX proteins in the PDB [71]. The most similar 3D structures to both proteins are those of *E. coli* T4 phage protein UvsX and hyperthermophilic *Thermotoga maritima* RecA [69, 72]. Superposition of T4 UvsX on UvsX_t_ resulted in an RMSD of 2.0 Å (246 residues), whereas UvsX_p_ superimposed with an RMSD of 1.8 Å (238 residues) (Fig. 13). The structures align very well, but the flexible N-terminal domain is missing in T4 UvsX. The superposition of hyperthermophilic *T. maritima* RecA on the UvsX structures (Fig. 13B) gives RMSD values of 3.0 Å (242 residues) for UvsX_t_ and 3.1 Å (237 residues) for UvsX_p_. Most of the secondary structural elements align well, but the N-terminus contains an extended α-helix 1 in *Tm*RecA and shows a distinct different orientation. This is similar to *E. coli* RecA, which is very well described, with 12 x-ray structures and 5 EM structures deposited in the PDB [11, 73, 74]. The PDB entry 2REB of *Ec*RecA superposes with an RMSD of 1.45 Å for 297 residues with *Tm*RecA [56]. The UvsX structures align well with this *Ec*RecA structure, with RMSDs of 2.6 Å (243 residues) for UvsX_t_, 2.7 Å (236 residues) for UvsX_p_, and 2.9 Å (234 residues) for T4 UvsX. Some structural differences to the new UvsX structures that are present in *Tm*RecA are also observed in *Ec*RecA: the helix α1, as well as extended β-sheets (β8, β9). Some features that are disordered in both our UvsX structures and T4 UvsX are instead visible in the *Tm*RecA structure. These structural parts are the two ssDNA binding loops, L1 and L2, which are usually only visible in the activated state of the recombinase filaments. Both T4 UvsX and *Tm*RecA have extended C-terminal β-sheets (β13, β14) compared to our UvsX structures.

The mechanism of recombination has been studied in detail on *Ec*RecA to dissect the protein, ssDNA, and dsDNA interactions in pre- and postsynaptic filaments [10, 53]. Below, we compare the UvsX structures presented here with those of *Ec*RecA, T4 UvsX, and *Tm*RecA (Fig. 13A and B). In the sequence alignment of selected recombinases, essential regions in the recombinase sequences are highlighted (Fig. 1). The ATP binding site (Walker A box or P-loop) is conserved in our UvsX proteins (residues 61–68, corresponding to residues 67–74 in *Ec*RecA) and adopts the consensus sequence [G/A]xxxxGK[T/S]. This region is present in all three structures, between β-sheet 3 and α-helix 3, and overlaps in a superposition of the three-dimensional structures. The ssDNA binding site includes the flexible loops L1 (residues 161–170, corresponding to residues 157–166 in *Ec*RecA) and L2 (residues 202–216 (UvsX_t_)/215 (UvsX_p_), corresponding to residues 197–211 in *Ec*RecA), which are disordered in the here described UvsX structures. These loops are usually only visible in crystal structures of ssDNA-bound forms. An exception is the structure of *Tm*RecA, where L1 and L2 are ordered. The Mg^2+^ binding motifs, T68 in the ATP binding site of our UvsXs (corresponding to T74 in *Ec*RecA) and the Walker B box (four hydrophobic residues, followed by aspartic acid, residues 141–145 in *Ec*RecA) are also structurally conserved in the UvsX_t_ and UvsX_p_ sequences (residues 145–149), but no Mg^2+^ was observed in the crystal structures, as it was not added during protein production or crystallization. Furthermore, the hydrolytic E93 (E97 in *Ec*RecA) and the [K,R]x[K,R] region (residues 249–251 in *Ec*RecA), which activate the attacking water molecule for ATP hydrolysis are also found in the UvsX protein sequences (residues 256–258 in UvsX_t_ and 253–255 in UvsX_p_) and can be found in the identical location.

A superposition of UvsX_t_ and UvsX_p_ on the cryo Electron Microscopy structure of DNA-bound active *E. coli* filament (7JY6) reveals that E93 in our UvsX structures is in the same position as the catalytic E96 in *Ec*RecA, as well as E92 in T4 UvsX and E98 in *Tm*RecA. The loop where this catalytic residue is situated faces in the *E. coli* EM structure towards the previous RecA molecule in the filament. The residues K249 and K251 of the hydrolytic motif [K,R]x[K,R] in the *Ec*RecA filament are in the same position as K256/R258 in UvsX_t_, as well as K253/R255 in UvsX_p,_ and K246/R248 in T4 UvsX, and K250/K252 in *Tm*RecA. These residues in the *Ec*RecA filament face the Mg^2+^ and ATP-γ-S bound at the RecA-RecA interface in the EM structure and thus interact with the next RecA molecule in the filament. The ATP-binding Walker motif and the Mg^2+^ binding aspartate residue superpose well in the discussed structures here. This means the hydrolytic mechanism in the new UvsX recombinases should be very similar to that in the known structures.

**Figure 14.**
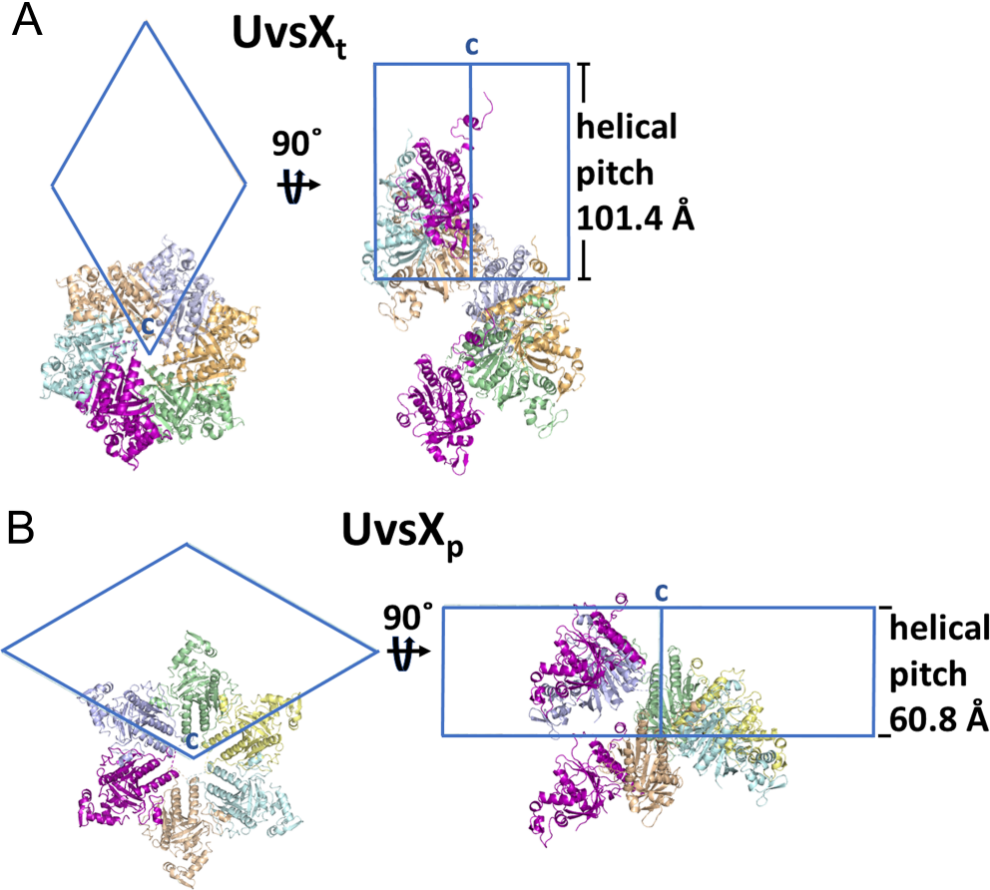
Crystal packing of UvsX_t_ (**A**) and UvsX_p_ (**B**) .The helical pitch of the filaments coincide with the crystallographic c-axis. The pitch in UvsX_t_ is 101.4 Å and 60.8 Å in UvsX_p_.

The crystal structures of both UvsX recombinases are in the crystallographic space group P6_1_, and the symmetry related molecules in the unit cell form a helical filament (coinciding with the crystallographic c-axis). The pitch in UvsX_t_ is 101.4 Å and 60.8 Å in UvsX_p_ (Fig. 14). This crystallographic filament can be compared with other recombinase filament structures. Recombinase helical filaments are typically highly flexible to allow the stretching of DNA. In *Ec*RecA, x-ray structures pitch values of 74–83 Å for the unbound compressed helical filaments are reported, whereas in the presence of ssDNA, the observed pitches in the active filament are 92–95 Å [10, 73, 74]. In archaeal RadA pitch values from 90 Å (PDB 2GDJ) to 107–109 Å (PDB 3ETL) are observed in *Methanococcus* species crystal structures [75, 76]. These values are high considering that neither structure have DNA bound. Despite the absence of bound DNA, yeast Rad51 shows in its crystal structure a very stretched filament with a length of 130 Å (PDB 3LDA) [9]. However, crystallographic filaments may not accurately reflect the DNA binding conformations. To learn more about the mechanism of the phage UvsX proteins described here, ssDNA-bound structures would be necessary.

Although the UvsX_t_ and UvsX_p_ overall display quite similar structure compared to *Ec*RecA, the viral enzymes cannot compensate for the loss of the *recA* gene. These findings are not surprising, considering the low sequences identities of 27% or less. The residues on the surfaces of the proteins, as well as the loops and termini that are responsible for protein–protein interactions, are even less conserved than the DNA recognition motifs. Therefore, neither UvsX_t_ nor UvsX_p_ can substitute for *Ec*RecA in the machinery required for UV-damage response.

Enzymatic assays for the novel recombinases UvsX_t_ and UvsX_p_ confirm DNA-binding and strand-displacement activities. UvsX_t_ and UvsX_p_ both have lower affinity for DNA than *Ec*RecA (*K*_d_ 210 nM) [77]. However, this may be common among viral recombinases, as T4 UvsX also has a lower affinity than RecA, with a *K*_d_ of 760 nM [8]. UvsX_t_ has a binding affinity close to T4 UvsX with a similar *K*_d_ of 741 nM; UvsX_p_ demonstrates tighter DNA binding with a *K*_d_ almost 300 nM less (469 nM). Interestingly, in contrast to the assays described here, those performed on T4 UvsX were conducted with ATP. Previous research into recombinases suggests that ATP is bound for ATP hydrolysis; however, UvsX does not bind ssDNA in the absence of ATP. Here, we show that the novel UvsX_t_ and UvsX_p_ proteins do not require ATP for DNA binding, demonstrating an advantageous property if solely being used to bind DNA to improve DNA stability. However, ATP is still required for strand displacement activity, with higher ATP concentrations leading to improved performance.

For use in biotechnological applications, proteins need to be stable across a range of conditions, including extremes of temperature. The stability of UvsX_t_ and UvsX_p_ was analysed using nanoDSF and TSAs. Results showed that both proteins are stable, but have different melting temperatures as expected. The *T*_m_ of UvsX_t_ is, depending on the conditions, up to 30°C higher than that of UvsX_p_, indicative of the different initial sampling environments of hot spring and the deep sea, respectively. Results from TSAs also indicated increased stability at pH 5–7 for UvsX_t_ (Supplementary Fig. S1), aligning with the conditions found in these environments (pH 5–9). For UvsX_p_, the stable pH range is 5–8 (Supplementary Fig. S2). Although the nanoDSF data matched the TSA data for UvsX_t_, there was a 10°C difference between the results for UvsX_p_ likely due to different starting buffer in the experiments; the buffer used for nanoDSF contained stabilizing components as identified in TSAs. UvsX_t_ is stable across several conditions, some of which increased the *T*_m_ to >70°C, and very few conditions screened had a negative effect on stability. In comparison, fewer conditions increased the stability of UvsX_p_, increasing the *T*_m_ to only >60°C. However, UvsX_p_ demonstrated greater stability over a wider range of chemical conditions, with only a few having a negative effect. With UvsX_t_ being able to function at higher temperatures and UvsX_p_ being stable over a wide range of chemicals, both enzymes are likely candidates for use in biotechnology.

Due to the SARS-CoV-2 pandemic and the increased interest in nucleic acid detection technologies, LAMP and its derivatives are garnering more attention. However, reduced sensitivity and specificity have been demonstrated in comparison to ‘gold standard’ PCR testing. In this study, we tested UvsX_t_ as an additive in RT-LAMP to assess its effects on specificity and sensitivity, as thermostability analysis indicated it would tolerate the assay temperature (65°C). In comparison, T4 UvsX has a melting temperature of ∼53°C and would be unsuitable for such an assay [78]. Similarly, *Ec*RecA is known to be fully denatured at 65°C. Here, UvsX_t_ acted solely as a DNA-binding protein, without strand-displacement activity, as ATP was not added to the reactions. Results indicate that UvsX_t_ can reduce the time taken to detect 10 ng of MS2 RNA, with this effect becoming more evident with increasing UvsX_t_ concentration (Fig. 7). RT-LAMP with the addition of 5 µM of UvsX_t_ then showed increased sensitivity in the detection of MS2 RNA (Fig. 8). These experimental results provide increasingly more valuable information for the improvement of LAMP assays, as these are fast and easy to use, and have proven invaluable during the recent SARS-CoV-2 pandemic.

## Conclusion

Here, we have described two novel phage recombinases, UvsX_t_ and UvsX_p_. Sequence alignments and crystal structures of both new recombinases are similar to *Ec*RecA, T4 UvsX, and *Tm*RecA, and thus we can assume similar mechanisms of function. However, the bacterial and viral enzymes are significantly different, and the viral recombinases cannot compensate for the loss of the *recA* gene to rescue *E. coli* knockouts. Both enzymes have demonstrated stability over a wide range of chemical conditions, with UvsX_t_ having a *T*_m_ of 71°C under stabilizing conditions. This highlights their potential use in biotechnological applications, where there is ever-increasing demand for new enzymes to drive progress. One such application is in RT-LAMP assays for nucleic acid detection, where we have shown that UvsX_t_ can improve both the sensitivity and speed of the assay. There is an optimistic prospect for the UvsXs enhancing other applications where recombinases are utilized, but there is a requirement for increased thermal stability or varied chemical conditions.

## Supplementary Material

gkag069_Supplemental_File

## Data Availability

The crystal structures of UvsX_t_ and UvsX_p_ have been deposited in the Protein Data Bank under accession numbers 7Z3M and 9GBG, respectively. All other data needed to evaluate the conclusions in the paper are present in the paper and/or Supplementary Data.

## References

[1] Kowalczykowski SC, Dixon DA, Eggleston AK et al. Biochemistry of homologous recombination in *Escherichia coli*. Microbiol Rev. 1994;58:401–65. 10.1128/mr.58.3.401-465.1994. 7968921 PMC372975

[2] Haldenby S, White MF, Allers T. RecA family proteins in archaea: radA and its cousins. Biochem Soc Trans. 2009;37:102–7. 10.1042/BST0370102. 19143611

[3] Del Val E, Nasser W, Abaibou H et al. RecA and DNA recombination: a review of molecular mechanisms. Biochem Soc Trans. 2019;47:1511–31. 10.1042/BST20190558. 31654073

[4] Kowalczykowski SC, Bianco PR, Tracy RB. DNA strand exchange proteins a biochemical and physical comparison. Front Biosci. 1998;3:D570–603. 10.2741/A304. 9632377

[5] Chow SA, Chiu S-K, Wong BC. RecA protein-promoted homologous pairing and strand exchange between intact and partially single-stranded duplex DNA. J Mol Biol. 1992;223:79–93. 10.1016/0022-2836(92)90717-X. 1530979

[6] Gamper HB, Hou Y-M, Kmiec EB. Evidence for a four-strand exchange catalyzed by the RecA protein. Biochemistry. 2000;39:15272–81. 10.1021/bi001704o. 11106508

[7] Robu ME, Inman RB, Cox MM. RecA protein promotes the regression of stalled replication forks *in vitro*. Proc Natl Acad Sci USA. 2001;98:8211–8. 10.1073/pnas.131022698. 11459955 PMC37423

[8] Maher RL, Morrical SW. Coordinated binding of single-stranded and double-stranded DNA by UvsX recombinase. PLoS One. 2013;8:e66654. 10.1371/journal.pone.0066654 . 23824136 PMC3688935

[9] Chen J, Villanueva N, Rould MA et al. Insights into the mechanism of Rad51 recombinase from the structure and properties of a filament interface mutant. Nucleic Acids Res. 2010;38:4889–906. 10.1093/nar/gkq209. 20371520 PMC2919713

[10] Chen Z, Yang H, Pavletich NP. Mechanism of homologous recombination from the RecA–ssDNA/dsDNA structures. Nature. 2008;453:489–94. 10.1038/nature06971. 18497818

[11] Egelman EH, Stasiak A. Structure of helical RecA–DNA complexes. Complexes formed in the presence of ATP-gamma-S or ATP. J Mol Biol. 1986;191:677–97. 10.1016/0022-2836(86)90453-5. 2949085

[12] Yu X, Egelman EH. DNA conformation induced by the bacteriophage T4 UvsX protein appears identical to the conformation induced by the *Escherichia coli* RecA protein. J Mol Biol. 1993;232:1–4. 10.1006/jmbi.1993.1363. 8331653

[13] Liu J, Morrical SW. Assembly and dynamics of the bacteriophage T4 homologous recombination machinery. Virol J. 2010;7:357. 10.1186/1743-422X-7-357. 21129202 PMC3016280

[14] Cai Y, Cheng T, Yao Y et al. *In vivo* genome editing rescues photoreceptor degeneration via a Cas9/RecA-mediated homology-directed repair pathway. Sci Adv. 2019;5:eaav3335. 10.1126/sciadv.aav3335. 31001583 PMC6469935

[15] Nozaki S . Rapid and accurate assembly of large DNA assisted by *in vitro* packaging of bacteriophage. ACS Synth Biol. 2022;11:4113–22. 10.1021/acssynbio.2c00419. 36446634 PMC9764419

[16] Corbett SL, Sharma R, Davies AG et al. Enhancement of RecA-mediated self-assembly in DNA nanostructures through basepair mismatches and single-strand nicks. Sci Rep. 2017;7:41081. 10.1038/srep41081. 28112216 PMC5253629

[17] Shigemori Y . Multiplex PCR: use of heat-stable *Thermus thermophilus* RecA protein to minimize non-specific PCR products. Nucleic Acids Res. 2005;33:e126. 10.1093/nar/gni111. 16087733 PMC1183492

[18] Stefanska A, Kaczorowska A-K, Plotka M et al. Discovery and characterization of RecA protein of thermophilic bacterium *Thermus thermophilus* MAT72 phage Tt72 that increases specificity of a PCR-based DNA amplification. J Biotechnol. 2014;182–183:1–10. 10.1016/j.jbiotec.2014.04.015. 24786823

[19] Li J, Macdonald J, Von Stetten F. Review: a comprehensive summary of a decade development of the recombinase polymerase amplification. Analyst. 2019;144:31–67. 10.1039/C8AN01621F. 30426974

[20] Piepenburg O, Williams CH, Stemple DL et al. DNA detection using recombination proteins. PLoS Biol. 2006;4:e204. 10.1371/journal.pbio.0040204. 16756388 PMC1475771

[21] Sherrill-Mix S, Hwang Y, Roche AM et al. Detection of SARS-CoV-2 RNA using RT-LAMP and molecular beacons. Genome Biol. 2021;22:169. 10.1186/s13059-021-02387-y. 34082799 PMC8173101

[22] Lai MY, Abdul Hamid MH, Jelip J et al. Recombinase-aided loop-mediated isothermal amplification on human *Plasmodium knowlesi*. Am J Trop Med Hyg. 2024;110:648–52. 10.4269/ajtmh.23-0572. 38412548 PMC10993835

[23] Smith JD, Schlecht U, Xu W et al. A method for high-throughput production of sequence-verified dna libraries and strain collections. Mol Syst Biol. 2017;13:913. 10.15252/msb.20167233. 28193641 PMC5327727

[24] Dunn K, Chrysogelos S, Griffith J. Electron microscopic visualization of RecA–DNA filaments: evidence for a cyclic extension of duplex DNA. Cell. 1982;28:757–65. 10.1016/0092-8674(82)90055-1. 7046950

[25] Aevarsson A, Kaczorowska A-K, Adalsteinsson BT et al. Going to extremes—a metagenomic journey into the dark matter of life. FEMS Microbiol Lett. 2021;368:fnab067. 10.1093/femsle/fnab067. 34114607

[26] Freitag-Pohl S, Jasilionis A, Håkansson M et al. Crystal structures of the *Bacillus subtilis* prophage lytic cassette proteins XepA and YomS. Acta Crystallogr D Struct Biol. 2019;75:1028–39. 10.1107/S2059798319013330. 31692476 PMC6834076

[27] Jasilionis A, Plotka M, Wang L et al. AmiP from hyperthermophilic *Thermus parvatiensis* prophage is a thermoactive and ultrathermostable peptidoglycan lytic amidase. Protein Sci. 2023;32:e4585. 10.1002/pro.4585. 36721347 PMC9929850

[28] Ahlqvist J, Linares-Pastén JA, Jasilionis A et al. Crystal structure of DNA polymerase I from *Thermus* phage G20c. Acta Crystallogr D Struct Biol. 2022;78:1384–98. 10.1107/S2059798322009895. 36322421 PMC9629493

[29] Gabler F, Nam S, Till S et al. Protein sequence analysis using the MPI Bioinformatics Toolkit. Curr Protoc Bioinformatics. 2020;72:e108. 10.1002/cpbi.108. 33315308

[30] Wegerer A, Sun T, Altenbuchner J. Optimization of an *E. coli* L-rhamnose-inducible expression vector: test of various genetic module combinations. BMC Biotechnol. 2008;8:2. 10.1186/1472-6750-8-2. 18194555 PMC2254391

[31] Magnusson AO, Szekrenyi A, Joosten H et al. nanoDSF as screening tool for enzyme libraries and biotechnology development. FEBS J. 2019;286:184–204. 10.1111/febs.14696. 30414312 PMC7379660

[32] Bruce D, Cardew E, Freitag-Pohl S et al. How to stabilize protein: stability screens for thermal shift assays and nano differential scanning fluorimetry in the Virus-X Project. J Vis Exp. 2019;144. 10.3791/58666. 30799847

[33] Grøftehauge MK, Hajizadeh NR, Swann MJ et al. Protein–ligand interactions investigated by thermal shift assays (TSA) and dual polarization interferometry (DPI). Acta Crystallogr D Biol Crystallogr. 2015;71:36–44. 10.1107/S1399004714016617. 25615858 PMC4304684

[34] Mueller AM, Breitsprecher D, Duhr S et al. MicroScale thermophoresis: a rapid and precise method to quantify protein–nucleic acid interactions in solution. In: Kaufmann M., Klinger C., Savelsbergh A. (eds), Functional Genomics, Methods in Molecular Biology. Springer New York, New York, NY, 2017, Vol. 1654, pp.151–64. 10.1007/978-1-4939-7231-9_10 28986788

[35] Kaboev O, Luchkina L, Shalguev V et al. Improved RecA-assisted fluorescence assay for DNA strand exchange reaction. BioTechniques. 2006;40:736–8. 10.2144/000112195. 16774116

[36] Chander Y, Koelbl J, Puckett J et al. A novel thermostable polymerase for RNA and DNA loop-mediated isothermal amplification (LAMP). Front Microbiol. 2014;5:395. 10.3389/fmicb.2014.00395. 25136338 PMC4117986

[37] Garman E . ‘Cool’ crystals: macromolecular cryocrystallography and radiation damage. Curr Opin Struct Biol. 2003;13:545–51. 10.1016/j.sbi.2003.09.013. 14568608

[38] Allan DR, Collins SP, Evans G et al. Status of the crystallography beamlines at Diamond Light Source. Eur Phys J Plus. 2015;130:56. 10.1140/epjp/i2015-15056-x.

[39] Broennimann C, Eikenberry EF, Henrich B et al. The PILATUS 1M detector. J Synchrotron Rad. 2006;13:120–30. 10.1107/S0909049505038665. 16495612

[40] Kabsch W . Integration, scaling, space-group assignment and post-refinement. Acta Crystallogr D Biol Crystallogr. 2010;66:133–44. 10.1107/S0907444909047374. 20124693 PMC2815666

[41] Winter G . xia2 : an expert system for macromolecular crystallography data reduction. J Appl Crystallogr. 2010;43:186–90. 10.1107/S0021889809045701.

[42] Winn MD, Ballard CC, Cowtan KD et al. Overview of the CCP 4 suite and current developments. Acta Crystallogr D Biol Crystallogr. 2011;67:235–42. 10.1107/S0907444910045749. 21460441 PMC3069738

[43] Winter G, Waterman DG, Parkhurst JM et al. DIALS: implementation and evaluation of a new integration package. Acta Crystallogr D Struct Biol. 2018;74:85–97. 10.1107/S2059798317017235. 29533234 PMC5947772

[44] McCoy AJ, Grosse-Kunstleve RW, Adams PD et al. Phaser crystallographic software. J Appl Crystallogr. 2007;40:658–74. 10.1107/S0021889807021206. 19461840 PMC2483472

[45] Murshudov GN, Skubák P, Lebedev AA et al. REFMAC 5 for the refinement of macromolecular crystal structures. Acta Crystallogr D Biol Crystallogr. 2011;67:355–67. 10.1107/S0907444911001314. 21460454 PMC3069751

[46] Sammito M, Meindl K, De Ilarduya IM et al. Structure solution with arcimboldo using fragments derived from distant homology models. FEBS J. 2014;281:4029–45. 10.1111/febs.12897. 24976038

[47] Cowtan K . The Buccaneer software for automated model building. 1. Tracing protein chains. Acta Crystallogr D Biol Crystallogr. 2006;62:1002–11. 10.1107/S0907444906022116. 16929101

[48] Emsley P, Lohkamp B, Scott WG et al. Features and development of Coot. Acta Crystallogr D Biol Crystallogr. 2010;66:486–501. 10.1107/S0907444910007493. 20383002 PMC2852313

[49] Chen VB, Wedell JR, Wenger RK et al. MolProbity for the masses–of data. J Biomol NMR. 2015;63:77–83. 10.1007/s10858-015-9969-9. 26195077 PMC4577456

[50] McNicholas S, Potterton E, Wilson KS et al. Presenting your structures: the CCP 4 mg molecular-graphics software. Acta Crystallogr D Biol Crystallogr. 2011;67:386–94. 10.1107/S0907444911007281. 21460457 PMC3069754

[51] Altschul SF, Gish W, Miller W et al. Basic local alignment search tool. J Mol Biol. 1990;215:403–10. 10.1016/S0022-2836(05)80360-2. 2231712

[52] Berman H, Henrick K, Nakamura H. Announcing the worldwide Protein Data Bank. Nat Struct Mol Biol. 2003;10:980. 10.1038/nsb1203-980. 14634627

[53] Yang H, Zhou C, Dhar A et al. Mechanism of strand exchange from RecA–DNA synaptic and D-loop structures. Nature. 2020;586:801–6. 10.1038/s41586-020-2820-9. 33057191 PMC8366275

[54] Cox MM . The bacterial RecA protein as a motor protein. Annu Rev Microbiol. 2003;57:551–77. 10.1146/annurev.micro.57.030502.090953. 14527291

[55] Robert X, Gouet P. Deciphering key features in protein structures with the new ENDscript server. Nucleic Acids Res. 2014;42:W320–4. 10.1093/nar/gku316. 24753421 PMC4086106

[56] Story RM, Weber IT, Steitz TA. The structure of the *E. coli* recA protein monomer and polymer. Nature. 1992;355:318–25. 10.1038/355318a0. 1731246

[57] Van Loenhout MTJ, Van Der Heijden T, Kanaar R et al. Dynamics of RecA filaments on single-stranded DNA. Nucleic Acids Res. 2009;37:4089–99. 10.1093/nar/gkp326. 19429893 PMC2709578

[58] Alekseev A, Serdakov M, Pobegalov G et al. Single-molecule analysis reveals two distinct states of the compressed RecA filament on single-stranded DNA. FEBS Lett. 2020;594:3464–76. 10.1002/1873-3468.13922. 32880917

[59] Shan Q, Cox MM. RecA filament dynamics during DNA strand exchange reactions. J Biol Chem. 1997;272:11063–73. 10.1074/jbc.272.17.11063. 9111000

[60] Cox MM . Regulation of bacterial RecA protein function. Crit Rev Biochem Mol Biol. 2007;42:41–63. 10.1080/10409230701260258. 17364684

[61] Kowalczykowski SC . Initiation of genetic recombination and recombination-dependent replication. Trends Biochem Sci. 2000;25:156–65. 10.1016/S0968-0004(00)01569-3. 10754547

[62] Craw P, Balachandran W. Isothermal nucleic acid amplification technologies for point-of-care diagnostics: a critical review. Lab Chip. 2012;12:2469. 10.1039/c2lc40100b. 22592150

[63] Notomi T . Loop-mediated isothermal amplification of DNA. Nucleic Acids Res. 2000;28:E63. 10.1093/nar/28.12.e63. 10871386 PMC102748

[64] Augustine R, Hasan A, Das S et al. Loop-mediated isothermal amplification (LAMP): a rapid, sensitive, specific, and cost-effective point-of-care test for coronaviruses in the context of COVID-19 pandemic. Biology. 2020;9:182. 10.3390/biology9080182. 32707972 PMC7464797

[65] Ferruz N, Schmidt S, Höcker B. ProteinTools: a toolkit to analyze protein structures. Nucleic Acids Res. 2021;49:W559–66. 10.1093/nar/gkab375. 34019657 PMC8262690

[66] Moe E, Leiros I, Riise EK et al. Optimisation of the surface electrostatics as a strategy for cold adaptation of uracil-DNA N-glycosylase (UNG) from Atlantic Cod (*Gadus morhua*). J Mol Biol. 2004;343:1221–30. 10.1016/j.jmb.2004.09.004. 15491608

[67] Gorfe AA, Brandsdal BO, Leiros H-KS et al. Electrostatics of mesophilic and psychrophilic trypsin isoenzymes: qualitative evaluation of electrostatic differences at the substrate binding site. Proteins. 2000;40:207–17. 10.1002/(SICI)1097-0134(20000801)40:2<207::AID-PROT40>3.0.CO;2-U. 10842337

[68] Brandsdal BO, Smalås AO, Åqvist J. Electrostatic effects play a central role in cold adaptation of trypsin. FEBS Lett. 2001;499:171–5. 10.1016/S0014-5793(01)02552-2. 11418134

[69] Gajewski S, Webb MR, Galkin V et al. Crystal structure of the phage T4 recombinase UvsX and its functional interaction with the T4 SF2 helicase UvsW. J Mol Biol. 2011;405:65–76. 10.1016/j.jmb.2010.10.004. 21035462 PMC3006652

[70] Abramson J, Adler J, Dunger J et al. Accurate structure prediction of biomolecular interactions with AlphaFold 3. Nature. 2024;630:493–500., 10.1038/s41586-024-07487-w. 38718835 PMC11168924

[71] Madej T, Marchler-Bauer A, Lanczycki C et al. Biological assembly comparison with VAST+. In: Gáspári Z. (ed), Structural Bioinformatics, Methods in Molecular Biology. Springer US, New York, NY, 2020, Vol. 2112, pp.175–86. 10.1007/978-1-0716-0270-6_13 32006286

[72] Lee S, Kim TG, Jeong E-Y et al. Crystal structure of *Thermotoga maritima* RecA. PDB. 2010;

[73] Story RM, Steitz TA. Structure of the recA protein–ADP complex. Nature. 1992;355:374–6. 10.1038/355374a0. 1731253

[74] Xing X, Bell CE. Crystal structures of *Escherichia coli* RecA in complex with MgADP and MnAMP−PNP . Biochemistry. 2004;43:16142–52. 10.1021/bi048165y. 15610008

[75] Galkin VE, Wu Y, Zhang X-P et al. The Rad51/RadA N-terminal domain activates nucleoprotein filament ATPase activity. Structure. 2006;14:983–92. 10.1016/j.str.2006.04.001. 16765891

[76] Li Y, He Y, Luo Y. Conservation of a conformational switch in RadA recombinase from *Methanococcus maripaludis*. Acta Crystallogr D Biol Crystallogr. 2009;65:602–10. 10.1107/S0907444909011871. 19465774 PMC2685736

[77] Gataulin DV, Carey JN, Li J et al. The ATPase activity of *E. coli* RecA prevents accumulation of toxic complexes formed by erroneous binding to undamaged double stranded DNA. Nucleic Acids Res. 2018;46:9510–23. 10.1093/nar/gky748. 30137528 PMC6182174

[78] Zhang L, Wang E, Wu L et al. Rational design of UvsX recombinase variants for enhanced performance in recombinase polymerase amplification applications. Biochemistry. 2025;64:2025–38. 10.1021/acs.biochem.5c00098. 40261914

