# Supplementary material for: Structure, function, and applications of two novel phage recombinases from extreme environments
Source: Nucleic Acids Res. 2026 Feb 11;54(4):gkag069. doi: 10.1093/nar/gkag069 (PMC12890299; doi:10.1093/nar/gkag069)
Supplement: gkag069_Supplemental_File [file gkag069_supplemental_file.pdf]

A

| | 1 | 2 | 3 | 4 | 5 | 6 | 7 | 8 | 9 | 10 | 11 | 12 |
|---|---|---|---|---|---|---|---|---|---|---|---|---|
| A | 61.0 | 61.1 | 51.4 | 50.1 | 55.5 | 60.4 | 50.2 | 55.4 | 58.7 | 52.2 | 56.7 | 59.6 |
| B | 53.3 | 57.4 | 60.8 | 52.5 | 58.3 | 60.4 | 51.4 | 55.9 | 59.3 | 60.4 | 61.7 | 61.8 |
| C | 61.6 | 61.9 | 62.1 | 61.8 | 61.9 | 62.2 | 57.3 | 59.1 | 60.3 | 61.1 | 61.9 | 61.9 |
| D | 60.7 | 61.2 | 61.9 | 60.6 | 61.4 | 61.8 | 59.7 | 59.7 | 60.8 | 59.8 | 60.6 | 61.2 |
| E | 62.3 | 62.1 | 62.0 | 60.8 | 61.4 | 61.8 | 59.4 | 59.7 | 59.7 | 60.1 | 60.9 | 60.9 |
| F | 60.2 | 60.7 | 61.0 | 61.0 | 61.4 | 61.4 | 61.5 | 61.5 | 61.1 | 61.3 | 61.2 | 60.6 |
| G | 61.1 | 61.4 | 60.6 | 61.5 | 61.3 | 61.0 | 61.4 | 61.4 | 60.5 | 60.5 | 58.7 | 57.3 |
| H | 58.5 | 58.4 | 57.6 | 58.2 | 55.9 | 54.0 | 60.0 | 59.2 | 58.4 | 56.8 | 50.5 | 47.3 |

B

| | 1 | 2 | 3 | 4 | 5 | 6 | 7 | 8 | 9 | 10 | 11 | 12 |
|---|---|---|---|---|---|---|---|---|---|---|---|---|
| A | 61.4 | 61.2 | 50.9 | 33.9<br>43.9 | 48.3 | 53.3 | 54.5 | 56.4 | 58.3 | 61.3 | 65.7 | 62.9 |
| B | 71.4 | 67.8 | 67.3 | 66.1 | 64.4 | 62.7 | 67.5 | 66.3 | 65.9 | 64.6 | 63.7 | 62.4 |
| C | 65.5 | 64.3 | 63.8 | 62.9 | 62.2 | 61.3 | 61.6 | 61.6 | 61.4 | 61.2 | 61.1 | 60.7 |
| D | 63.6 | 63.2 | 62.9 | 62.3 | 61.6 | 67.0 | 67.7 | 66.6 | 64.9 | 63.2 | 62.1 | 61.7 |
| E | 61.8 | 61.0 | 61.7 | 61.1 | 61.9 | 60.6 | 63.5 | 61.7 | 62.0 | 61.6 | 60.9 | 38.9<br>60.2 |
| F | 59.9 | 59.8 | 61.3 | 62.5 | 61.3 | 63.4 | 61.0 | 66.0 | 63.9 | 59.5 | 60.9 | 63.5 |
| G | 61.2 | 61.7 | 60.1 | 60.6 | 61.2 | 61.1 | 60.9 | 42.8 | 62.1 | 61.9 | 53.6 | 41.9 |
| H | 45.6 | 43.6 | 40.8 | 43.2 | 42.4 | 61.2 | 60.2 | 61.4 | 61.2 | 62.1<br>86.5 | 60.9 | 60.7 |

C

| | 1 | 2 | 3 | 4 | 5 | 6 | 7 | 8 | 9 | 10 | 11 | 12 |
|---|---|---|---|---|---|---|---|---|---|---|---|---|
| A | 61.1 | 61.1 | 50.8 | 63.8 | 63.1 | 62.1 | 61.5 | 60.6 | 64.1 | 61.4 | 62.0 | 60.9 |
| B | 63.3 | 61.4 | 61.9 | 61.2 | 62.7 | 60.9 | 61.4 | 60.8 | 62.9 | 61.3 | 61.8 | 60.9 |
| C | 61.7 | 61.1 | 61.9 | 61.4 | 61.8 | 61.3 | 61.7 | 61.0 | 59.1 | 60.2 | 60.4 | 60.9 |
| D | 32.5 | 61.1 | 61.1 | 61.3 | 60.0 | 61.2 | 60.9 | 61.3 | 61.4 | 61.1 | 61.7 | 61.2 |
| E | 64.6 | 61.9 | 61.4 | 61.3 | 61.4 | 61.2 | 28.5 | 61.2 | 61.2 | 61.2 | 45.2 | 61.3 |
| F | 61.4 | 61.3 | N/S | 59.0 | 60.0 | 61.3 | 60.6 | 61.3 | 60.1 | 61.1 | 60.4 | 61.1 |
| G | 45.5 | 45.8 | 61.7 | 61.1 | 61.7 | 61.3 | N/S | N/S | 59.8 | 57.9 | 58.7 | 61.2 |
| H | 61.9 | 61.2 | 61.8 | 61.2 | 61.7 | 61.4 | 62.2 | 61.3 | 62.2 | 61.2 | 60.9 | 61.0 |

**Figure S1. Results of TSA of UvsX$_t$.** (A) The pH screen, (B) salt, and (C) osmolyte. Screen compositions are available at Molecular Dimensions.

A

| | 1 | 2 | 3 | 4 | 5 | 6 | 7 | 8 | 9 | 10 | 11 | 12 |
|---|---|---|---|---|---|---|---|---|---|---|---|---|
| A | 39.8 | 40.2 | N/S | N/S | 28.0 | 30.5<br>36.6 | 30.0<br>89.5 | 32.4 | 37.9 | 90.5 | 27.5 | 35.2 |
| B | N/S | 32.0 | 41.3 | N/S | 30.0<br>41.9 | 42.6 | N/S | 30.0<br>83.6 | 38.9 | 35.9 | 41.6 | 42.9 |
| C | 39.9 | 43.8 | 43.4 | 41.8 | 42.2 | 43.3 | 33.0 | 37.2 | 39.6 | 37.3 | 41.1 | 42.7 |
| D | 39.6 | 41.0 | 41.9 | 40.2 | 41.5 | 42.2 | 38.6 | 38.6 | 39.5 | 39.6 | 40.7 | 41.3 |
| E | 43.1 | 43.6 | 43.0 | 41.6 | 42.3 | 42.9 | 37.5 | 36.8 | 36.6 | 39.7 | 40.5 | 40.6 |
| F | 39.1 | 39.4 | 39.4 | 40.8 | 41.3 | 40.4 | 41.0 | 41.3 | 41.0 | 40.7 | 41.3 | 40.8 |
| G | 40.1 | 40.0 | 38.9 | 41.1 | 40.9 | 40.7 | 41.0 | 40.8 | 39.8 | 38.6 | 35.9 | 33.7 |
| H | 38.3 | 38.2 | 37.0 | 33.0 | 32.0 | 31.0 | 40.0 | 38.9 | 37.2 | 34.9 | 30.0 | 30.0 |

B

| | 1 | 2 | 3 | 4 | 5 | 6 | 7 | 8 | 9 | 10 | 11 | 12 |
|---|---|---|---|---|---|---|---|---|---|---|---|---|
| A | 40.2 | 40.2 | N/S | 82.1 | 61.0<br>75.7 | 43.1<br>58.2 | N/S | 33.5 | 37.0 | 40.2 | 52.8 | 46.3 |
| B | 64.3 | 59.2 | 54.9 | 51.8 | 48.0 | 44.3 | 61.9 | 55.9 | 53.1 | 50.7 | 48.9 | 45.3 |
| C | 48.7 | 46.1 | 45.1 | 44.7 | 42.9 | 41.8 | 43.6 | 42.9 | 42.3 | 41.9 | 41.4 | 40.8 |
| D | 50.8 | 51.5 | 49.2 | 47.8 | 45.3 | 59.5 | 57.3 | 54.4 | 50.4 | 46.6 | 43.1 | 41.4 |
| E | 41.7 | 40.8 | 43.0 | 41.4 | 42.3 | 40.8 | 45.7 | 41.6 | 41.9 | 41.6 | 40.8 | 38.8 |
| F | 42.0 | 36.1 | 40.4 | 41.6 | 40.7 | 38.8 | 39.0 | 38.5 | 39.8 | 40.9<br>56.3 | 40.4 | 39.2 |
| G | 41.7 | 28.5 | 28.0 | 37.2 | 40.5 | 41.0 | 40.1 | 44.5 | 43.0 | 42.9 | 42.4 | 41.9 |
| H | 42.2 | 41.9 | 42.2 | 43.0 | 43.1 | 40.4 | 39.6 | 40.5 | 40.4 | 40.3 | 40.4 | 40.2 |

C

| | 1 | 2 | 3 | 4 | 5 | 6 | 7 | 8 | 9 | 10 | 11 | 12 |
|---|---|---|---|---|---|---|---|---|---|---|---|---|
| A | 39.9 | 40.0 | 27.5 | 43.9 | 43.1 | 42.0 | 41.1 | 40.1 | 45.4 | 41.5 | 43.0 | 40.1 |
| B | 44.0 | 40.4 | 42.3 | 40.1 | 43.8 | 40.2 | 40.5 | 40.1 | 43.2 | 41.0 | 41.5 | 40.2 |
| C | 42.6 | 39.9 | 42.2 | 40.2 | 41.1 | 41.1 | 41.9 | 39.6 | 33.2 | 39.4 | 38.5 | 39.8 |
| D | 39.2 | 39.6 | 39.6 | 40.1 | 39.8 | 40.2 | 37.4 | 39.7 | 40.2 | 39.8 | 40.5 | 40.0 |
| E | 50.8 | 41.9 | 41.8 | 40.1 | 41.4 | 40.2 | 35.9 | 40.2 | 40.6 | 40.0 | 90.0 | 39.9 |
| F | 39.2 | 39.9 | N/S | 42.6 | 40.7 | 40.2 | 38.1 | 40.3 | 37.6 | 39.8 | 38.0 | 40.3 |
| G | 39.0 | N/S | 41.2 | 40.3 | 41.9 | 40.1 | 75.3 | N/S | 38.0 | 35.4 | 39.9 | 40.1 |
| H | 40.7 | 40.1 | 43.1 | 40.7 | 42.0 | 40.5 | 42.7 | 40.6 | 42.6 | 40.5 | 39.6 | 39.9 |

**Figure S2. Results of TSA of UvsX$_p$.** (A) The pH screen, (B) salt, and (C) osmolyte. Screen compositions are available at Molecular Dimensions.

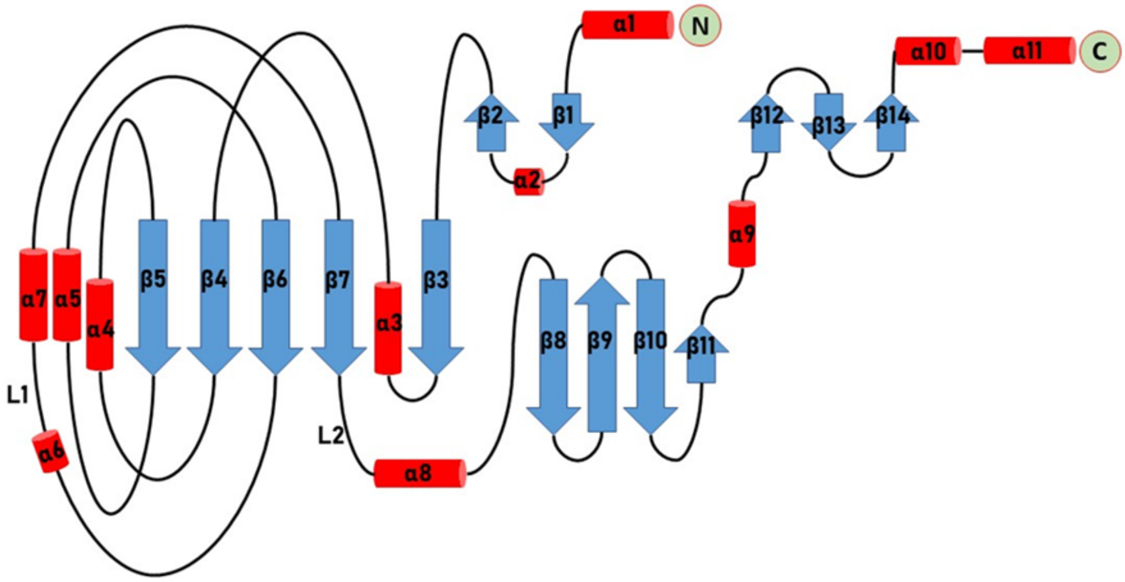


**Figure S3. Cartoon representation of $UvsX_p$ and $UvsX_t$.** Secondary structures are labelled, with α-helices in red and β-sheets in blue.
